# Genomic elucidation of hybridization between Liberica and excelsa coffee and its implications for coffee crop development

**DOI:** 10.1038/s41598-026-49305-5

**Published:** 2026-05-14

**Authors:** Aaron P. Davis, Amelia Shepherd-Clowes, Kenny Wee Ting Lee, Diana M. Jitam, Alasdair Clayre, Akshay Dashrath, Juan Viruel

**Affiliations:** 1 Royal Botanic Gardens, Kew, TW9 3AE, UK; 2 Earthlings Coffee, Kuching, Sarawak Malaysia; 3 Agriculture Research Centre Semonggok, KM20 Jalan Borneo Heights, 93250 Kuching, Sarawak Malaysia; 4 Institute of Borneo Studies, University of Malaysia Sarawak, 94300 Kota Samarahan, Sarawak Malaysia; 5 SICC Labs, Kodagu District, Suntikoppa, Karnataka 571237 India; 6 Technological College, University of Zaragoza, 22071 Huesca, Spain; 7 Institute for Biocomputation and Physics of Complex Systems (BIFI), University of Zaragoza, Mariano Esquillor, Edificio I+D, 50018 Zaragoza, Spain

**Keywords:** Genomics, Hybrids, Excelsa coffee, Liberica coffee, Malaysia, SNPs, Biotechnology, Genetics, Plant sciences

## Abstract

The development and establishment of a broader range of coffee species and hybrids is likely to play a key role in coffee farming sustainability in an era of accelerated climate change. We investigated hybridization between *Coffea liberica* (Liberica) and *C. dewevrei* (excelsa) utilizing 7,618 SNPs from 113 accessions, sampled across three continents. Our analyses demonstrated that these two species have hybridized readily in cultivation and produced fertile progeny with a wide range of genomic admixture. We revealed extensive genomic admixture in farmed accessions from Sarawak. The identified hybrids exhibited intermediate characteristics and overlapping values for key agronomic traits, including seed size and parchment thickness. These traits, which influence yield,  outturn and post-harvest processing, can be transferred via hybridization. The hybrids also have the potential to broaden the climate envelope for successful coffee cultivation and transfer disease resistance. Improved genotypes resulting from hybrids between *C. liberica* and *C. dewevrei* could be brought into production relatively quickly, as a means of developing both species. We propose the formal name *Coffea* × *libex* for the interspecies hybrid.

**Supplementary Information:**

The online version contains supplementary material available at 10.1038/s41598-026-49305-5.

## Introduction

The development and establishment of a broader range of coffee crop species and hybrids is likely to become a key requirement for achieving coffee farming sustainability in an era of accelerated climate change^1–3^. Future coffee crops will need to be commercially viable across the value chain, and operate under elevated temperatures, a broader range of annual precipitation patterns, including changes in timing and duration, and increasing frequency and severity of extreme weather events. Arabica (*Coffea arabica*) and robusta coffee (*C. canephora*) currently account for more than 99.99% of the world’s coffee^4^ and are not expected to require substantial replacement or intervention at a global level over the short-term^5^. However, as climate change progresses, the development and deployment of alternative, climate-appropriate coffee crops is required for communities where Arabica and robusta farming are already faltering or failing. Given the limitations for agricultural operability of Arabica and robusta under certain climate parameters, an expanded and diversified coffee portfolio will need to include additional *Coffea* species, to introduce the traits (and numerous genes) that are required for meaningful climate resileince^1–3^. Some diversification is already underway, with increased utilization of once more commonly grown species, including Liberica (*C. liberica*) and excelsa (*C. dewevrei*)^6^, Congo (*C. congensis*), and to a lesser extent Ibo/Zanzibar (*C. zanguebariae*), racemosa (*C. racemosa*), and Nandi (*C. eugenioides*) coffees^3,4,6–9^. Sierra Leone coffee (*C. stenophylla*) has recently attracted attention due to its ability to produce high-quality coffee, with an Arabica-like flavour, at substantially higher temperatures than Arabica, and under a longer dry season than robusta^2,10,11^. Although stenophylla coffee is not yet established within the value chain, it has become a focal species for the development of a climate resilient alternative to *C. arabica*.

Interspecies hybrids, other than the well-known hybrid *C. arabica* × *C. canephora*, are generally rare in coffee farming. However, hybridization has been utilised in several breeding contexts. In India crosses and backcrosses involving *C. canephora* × *C. congensis* have been developed and are in use^3,12,13^, as well as *C. arabica* × *C. liberica*^14^. In Brazil, breeding programmes have used *C. arabica* × *C. racemosa* hybrids^15–17^. Other interspecies hybrids have been reported, including *C. canephora* × *C. liberica*^18^, *C. liberica* × *C. eugenioides*^19,20^, and *C. canephora* × *C. racemosa*^12,21^.

Recent genomic and morphological research has clarified species delimitation within *C. liberica sensu lato*, demonstrating that it comprises three distinct species: *C. liberica* (Liberica), *C. dewevrei* (excelsa) and *C. klainei*^4^. Indigenous populations of *C. liberica* and *C. dewevrei* have discrete, geographical distributions and climate profiles, and possess distinct morphological and physiological traits relevant to crop development^4^. *Coffea dewevrei* possesses several attributes favourable for coffee production, including more flowers and fruits per branch/node, a smaller fruit, a thinner fruit pulp (mesocarp), thinner parchment (endocarp; i.e. the hard, crisp coating that surrounds the seed) and smaller Arabica-sized seeds, compared with *C. liberica*. These traits translate into higher fresh fruit yields, the facilitation of faster post-harvest processing, including reduced drying times, and improved outturns, relative to *C. liberica*. Outturn is defined as the conversion ratio of fresh fruit to clean coffee, which is a key factor in coffee farming profitability. Favourable traits for *C. liberica* include tolerance to high temperatures and successful cultivation across a broader range of precipitation regimes, including environments characterised by high rainfall and low elevations^22^. *Coffea liberica* may also tolerate longer and more severe dry periods than *C. dewevrei*^4,22^. Both wild and cultivated *C. liberica* demonstrate substantially greater climatic tolerance compared with *C. arabica*, particularly with respect to temperatures and precipitation. This information suggests that hybridization between *C. liberica* and *C. dewevrei* may provide opportunities to combine favourable agronomic and climatic characteristics within a single crop lineage.

The aim of this study is to verify and quantify hybridization between *C. liberica* and *C. dewevrei*, and to provide an initial assessment of its potential value for coffee crop development. Although hybrids between these two species have been reported^23^ and inferred^4^ based on morphology, their existence has not been confirmed by molecular data. Fruit and seed traits, specifically parchment (endocarp) thickness and seed dimensions, provide useful morpho-taxonomic markers for *C. liberica* and *C. dewevrei*^4^. Intermediate values for these characters have been observed in cultivated accessions, including farmed plants, suggesting the existence of interspecies hybrids. Hybridization has also been inferred in cultivated populations exhibiting high variability in leaf size and shape; floral traits, including flower number (per node), flower size and shape, and corolla lobe number (per flower); and fruit size, shape and colour, and pulp (mesocarp) thickness. *Coffea liberica* and *C. dewevrei* do not overlap in their wild distributions^4^. Consequently, this study is focused on cultivated plants and farmed populations.

Our study has five main objectives: (1) to confirm the existence of hybridization between *C. liberica* and *C. dewevrei*; (2) to estimate the frequency of hybridization between these species; (3) to quantify levels of genetic admixture in hybrid individuals; (4) to assess the influence of hybridization on morphology, with emphasis on seed and parchment traits of agronomic relevance; and (5) to evaluate the potential usefulness of hybridization and introgression for the breeding and improvement of *C. liberica* and *C. dewevrei*.

To identify hybrids we employed genomic data generated using the Angiosperms353 target capture kit^24–26^ analysing 7,618 SNPs from the exon regions^27^ across 113 accessions, sampled from three continents. The dataset includes predominantly cultivated material from farms and germplasm collections, with wild accessions used as reference standards. Morphological data on parchment (endocarp) thickness and seed dimensions were analysed to evaluate the phenotypic consequences of hybridization for these agronomic traits.

## Results

### Angiosperms353 data recovery

Across all accessions, we recovered between 72.4 and 91.1% (mean 87.4%) of the Angiosperms353 genes length, representing 208,305–262,158 (mean 251,364) bp per sample. Overall, there were 2.22% missing data in the alignments.

### SNP analyses

**Fig. 1:**
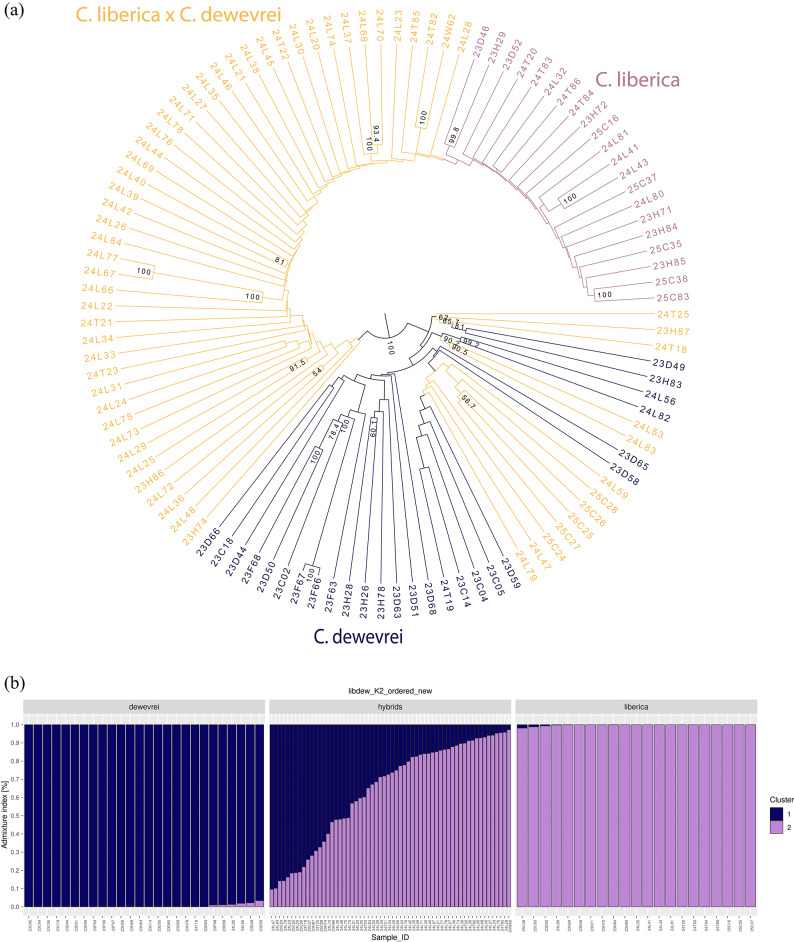

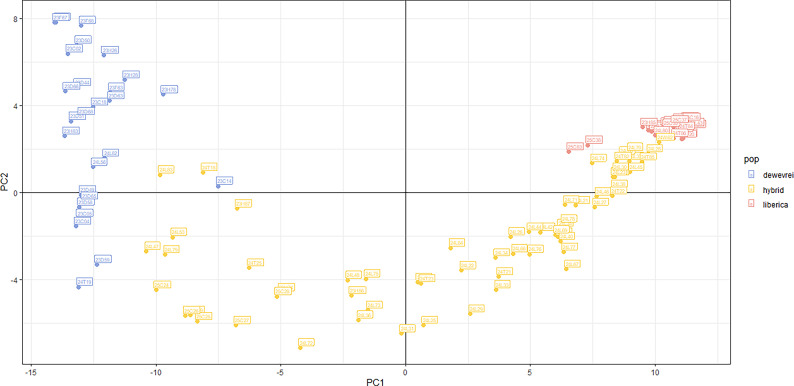
Genetic structure and relationships between and within *C. liberica* (Liberica), *C. dewevrei* (excelsa) and *C. liberica* × *C. dewevrei*. Based on 7,618 exon region SNPs. **a**, A genetic distance phylogenetic tree reconstructed with pairwise genetic distances (the proportion of loci that are different). The figures above branches indicate the BS values (BS values < 50 not shown). **b**, STRUCTURE^28^ analysis with the *K* value set to *K* = 2 to match the number of parental species, representing two clusters (*C. liberica*, *C. dewevrei*) and admixture accessions (hybrids). See Supplementary Fig. S1 for alternative *K* value analyses, and Supplementary Table S3 for *K* = 3 and *K* = 4 admixture values. **c**, PCoA analysis. PC1 (85.3% variance) separates the two parental species, with intermediate admixture accessions (hybrids). Accession information is provided in Supplementary Table S1.

To investigate genetic structure and relationships between *C. liberica*, *C. dewevrei* and suspected hybrids we analysed 7,618 SNPs derived from exon regions^27^ across 113 accessions (Supplementary Table S1). Evaluation of cluster number using Δ*K* supported *K* = 2 as the most likely value. STRUCTURE^28^ results for *K* = 2 are shown in Fig. 1b, with corresponding admixture proportions provided in Supplementary Table S2. STRUCTURE results for *K = 3* and *K = 4* are presented in Supplementary Fig. S1; with admixture values given in Supplementary Table S3.

At *K = 2*, no genomic admixture was detected for wild-sourced accessions of either *C. liberica* or *C. dewevrei*. In contrast, genomic admixture was detected in 60 cultivated samples. Of these, 40 samples showed admixture proportions of 56.9–97.4% from *C. liberica*, while 20 samples showed admixture proportions of 51.1–90.4% from *C. dewevrei* (Supplementary Table S2). Six cultivated accessions identified as *C. dewevrei* showed low levels of admixture from *C. liberica*, ranging from 1 to 3%, including samples from Sierra Leone (1% admixture, sample 23F68), Hawaii (1.1%, 23D58 and 3.4%, 23D59), India (1.9%, 24L56 and 1.3%, 24L82), and Costa Rica (2.2%, 23D49), as detailed in Supplementary Table S2. Similarly, three cultivated accessions identified as *C. liberica* showed low admixture from *C. dewevrei* ranging from 0.7 to 2%, originating from Dominica (1.9% admixture, sample 25C38), Cameroon (1.2%, 25C83), and Peninsula Malaysia (0.7%, 23D52).

Principal coordinate analysis (PCoA) (Fig. 1b) showed a clear separation between *C. liberica* and *C. dewevrei* along PC1, which explained 85.3% of the total variance. Admixture samples were positioned between the two parental clusters, with PC2 accounting for an additional 13% of variance. Two cultivated accessions of *C. liberica* from Dominica and Cameroon (25C38 and 25C83, respectively) were outliers relative to the otherwise tightly clustered *C. liberica* group, consistent with low levels of detected admixture.

A genetic distance-based phylogenetic tree reconstructed from pairwise genetic distances (Fig. 1a) supported the division of two principal groups. One comprised *C. dewevrei* and accessions with high proportions of *C. dewevrei* admixture, while the other comprised *C. liberica* and samples with high proportions of *C. liberica* admixture. Bootstrap support values across the tree were generally low.

### SNP analyses for Malaysia

**Fig. 2:**
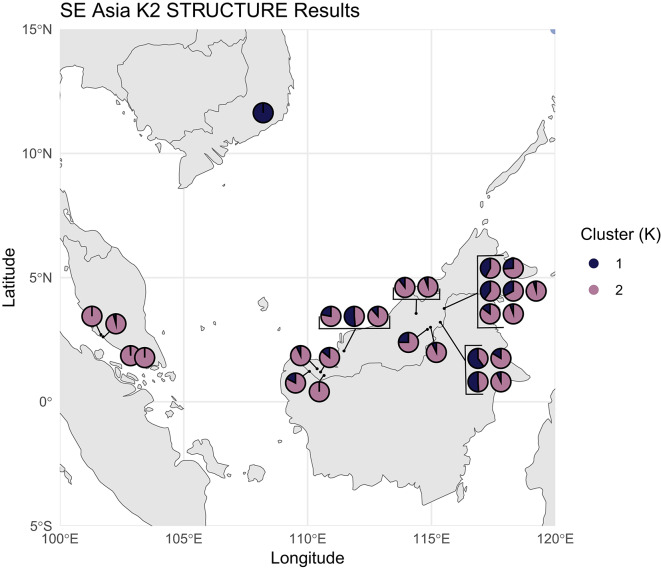
Admixture pie-charts for *C. liberica* (Liberica), *C. dewevrei* (excelsa) and *C. liberica* × *C. dewevrei*, for Vietnam, and Malaysia accessions. Admixture based on STRUCTURE^28^ analysis at *K* = 2 (see Fig. 1b) for 26 samples from Malaysia, with one representative sample of *C. dewevrei* from Vietnam. See Supplementary Tables S1, S4 for accession information, and Supplementary Table S2 for *K* = 2 values.

STRUCTURE^28^ analysis revealed a particularly high incidence of *C. liberica*-*C. dewevrei* admixture among farmed *C. liberica* from Sarawak (Fig. 2). At *K* = 2, admixture proportions ranged from 2.8 to 59.9% *C. dewevrei*. Of the 45 Sarawak accessions labelled as Liberica, 40 showed more than 2.8% *C. dewevrei* admixture, and 28 exceeded 12% admixture, with a maximum of 59.90% admixture. Only five accessions were identified as genetically ‘pure’ *C. liberica* (< 1% *C. dewevrei* admixture). In contrast, of the eight accessions from Peninsular Malaysia, six were genetically ‘pure’ *C. liberica* (< 1% admixture), and two showed low admixture levels of 4.4% and 8.6% (Supplementary Table S4).

Outside Malaysia, admixture was also detected in cultivated accessions from multiple regions. Four accessions from Indonesia showed admixture values of 13.6%, 51.9%, 85.7% and 90.4% *C. dewevrei*. Nine accessions from India exhibited admixture values between 34.8 and 89.8%. Four accessions from Costa Rica showed admixture ranging from 64.1 to 78%, and a single accession from Uganda exhibited 53.4% *C. dewevrei* admixture.

### Morphological analysis of seed and parchment

**Fig. 3:**
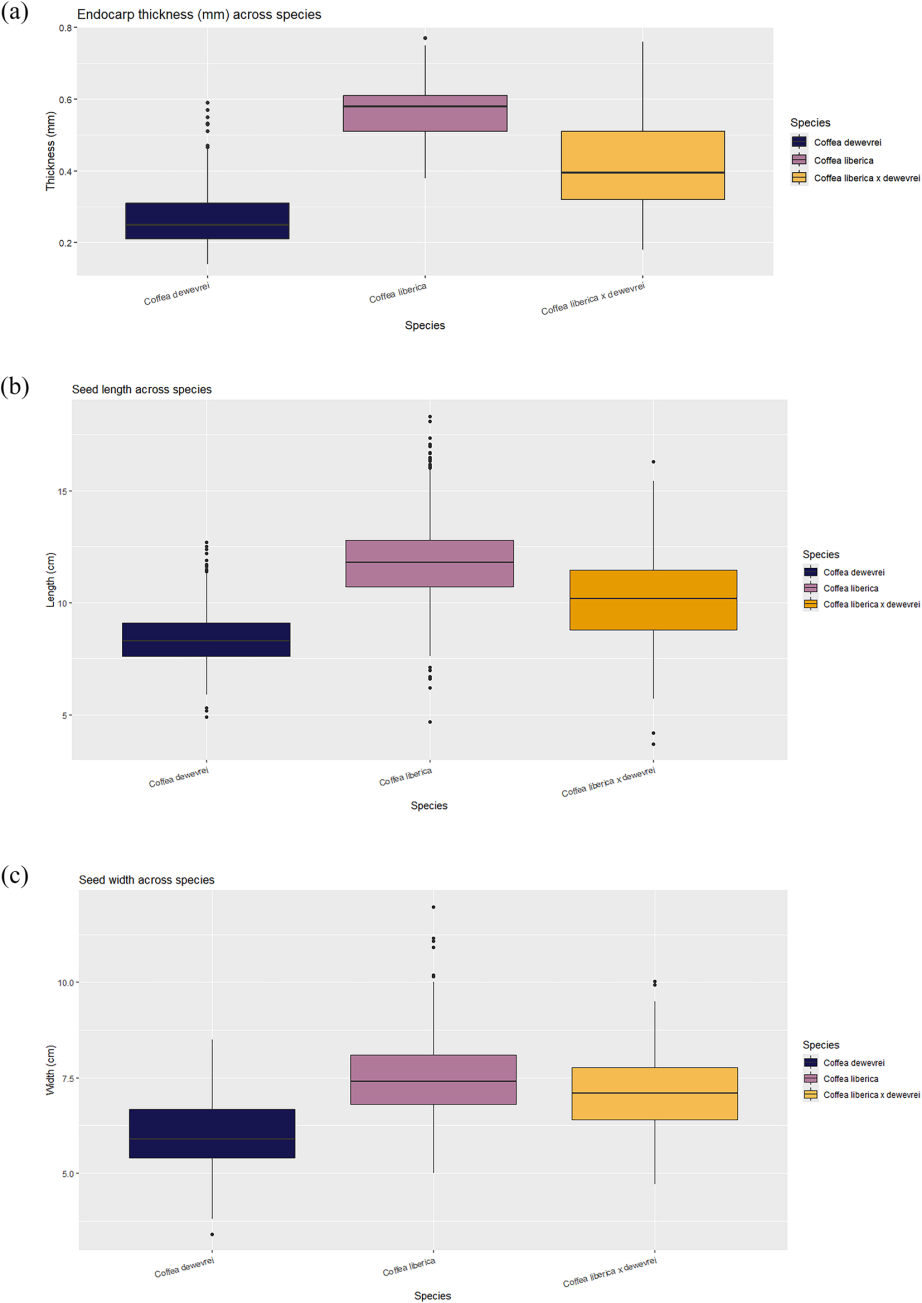
Box and whisker plots for parchment (endocarp) thickness and seed length and width for *C. liberica* (Liberica), *C. dewevrei* (excelsa) and *C. liberica* × *C. dewevrei.* Mean values in parentheses; quartile values are given in Supplementary Table S5.** a** Parchment thickness (mm): *C. liberica* (0.59 mm), *C. dewevrei* (0.27 mm) and *C. liberica* × *C. dewevrei* (0.44 cm). **b** Seed length (mm): *C. liberica* (11.85 mm), *C. dewevrei* (8.39 mm) and *C. liberica* × *C. dewevrei* (10.27 mm). **c** seed width (mm): *C. liberica* (7.51) mm), *C. dewevrei* (6.02 mm) and *C. liberica* × *C. dewevrei* (7.15 mm). See Supplementary Table S5 for descriptive statistics and details of ANOVA and TurkeyHSD test.

Seed size and parchment thickness differed markedly among *C. liberica*, *C. dewevrei*, and admixed accessions. For *C. dewevrei*, seed length and seed width measurements were 4.9–12.7 and 3.4–8.5 mm, respectively, with mean values of 8.4 × 6 mm. For *C. liberica*, seed length and seed width were 6.2–18.3 and 5.0–12.0 mm, with mean values of 11.7 × 7.7 mm. Admixed accessions spanned a broader range for seed length and seed width, of 3.4–16.7 and 4.7–10 mm, with mean values of 10.5 × 7.1 mm. Parchment thickness measurements for *C. dewevrei* were 0.14–0.59 mm, with a mean of 0.27 mm. For *C. liberica*, parchment thickness was 0.43–0.77 mm, with a mean of 0.59 mm. Admixture accessions showed intermediate and overlapping values for parchment thickness of 0.18–0.77 mm, with a mean of 0.44 mm. One way ANOVA and TukeyHSD tests showed that *C. liberica*, *C. dewevrei* and *C. liberica*, × *C. dewevrei* differed significantly for parchment thickness, seed length and seed width (Fig. 3, Supplementary Fig. S2, Supplementary Table S5 (with *p*-values given)).

**Fig. 4:**
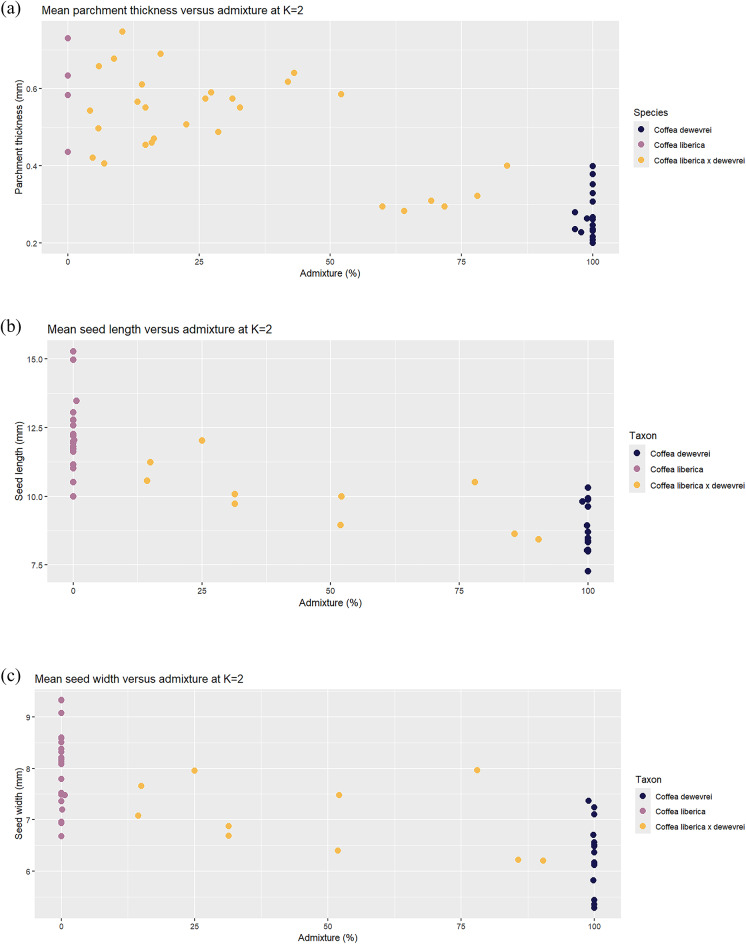
Mean parchment (endocarp) and seed measurements versus admixture at *K* = 2 for *C. liberica*, *C. dewevrei* and *C. liberica* × *C. dewevrei*. a, parchment thickness. **b**, seed length. **c**, seed width. See Supplementary Tables S7, S8 for accession details and mean values.

Where corresponding genomic and morphological data were available, we plotted admixture proportions at *K* = 2 were against seed length, seed width and parchment thickness. Despite limited sample sizes, accessions with higher proportions of *C. dewevrei* admixture were associated with thinner parchment and smaller seeds (Fig. 4).

### Other observations

**Fig. 5:**
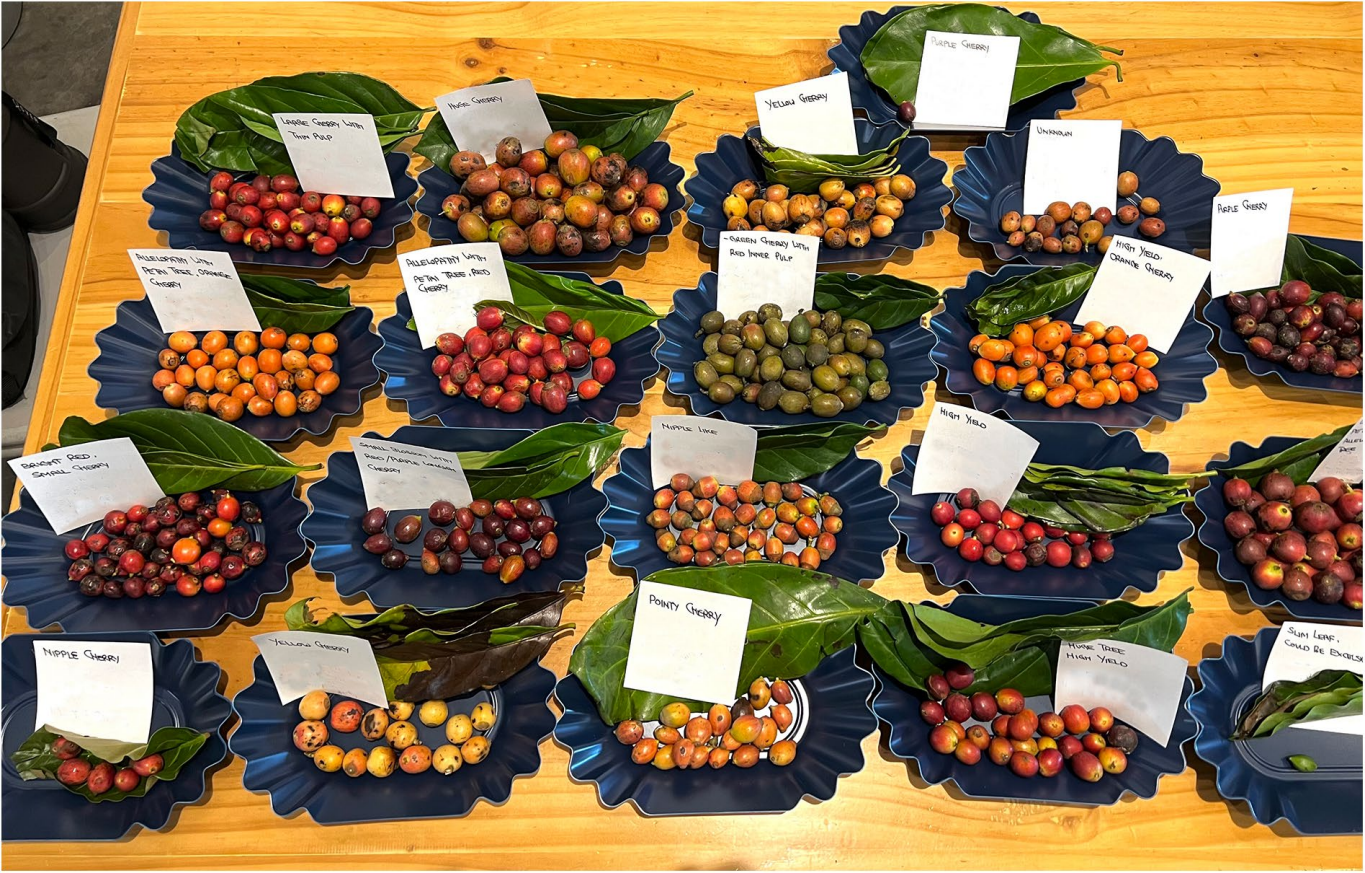
Variation in fruit size, shape and colour for *C. liberica* × *C. dewevrei* hybrids, from farms in the Upper Baram Valley, Sarawak. Image: Kenny Wee Ting Lee ©.

Field observations in Sarawak indicated that genotypes with high levels of *C. dewevrei* admixture (i.e. 50–75%) displayed combinations of parental traits. These plants, typically, exhibited more flowers and fruits per node/axil, and thinner fruit pulp (mostly mesocarp), compared with *C. liberica*. Leaf morphology ranged from those indistinguishable from *C. liberica*, to very large leaves characteristic of *C. dewevrei*^4^. Considerable variation was observed in flower size, shape and corolla lobe number, ranging from 5 to 12 lobes per flower. In *C. liberica*, individual flowers usually have 7–12 corolla lobes, and for *C. dewevrei* commonly 5 or sometimes 6. Fruit morphology among admixed plants was also highly variable (Fig. 5), including variation in fruit size, shape and colour. *Coffea liberica* fruits are typically red or rarely yellow, and *C. dewevrei* exhibits a wide range of colours including different shades and depths of yellow, orange, red, red-purple, and purple; highly admixed genotypes displayed especially diverse fruit colouration, including reds, oranges, and yellow, and purple (Fig. 5). Variation in nectary disc remains (the ‘woody’ area at the apex of the fruit) was observed across admixed and non-admixed populations alike. Large and small nectary disc remains are evident across admixture farm population, but this is also true of ‘pure’ wild and cultivated populations of *C. liberica* and *C. dewevrei*. Genotypes with low admixture of *C. dewevrei*, of around 10% or lower, tend to have a fruit and gross morphology that is identical to *C. liberica*, and vice versa.

Our measurement of 2,140 seeds (Supplementary Tables S7, S8) and observation of harvested seeds (coffee beans) across the full admixture spectrum, including those genotypes with around 50% admixture (at *K* = 2), showed negligible (< 5%) to small percentages (10%, rarely 20%) of fruits with a single seed. These single seeds are rounded in shape, rather than flat on one side (the adaxial surface) and are usually referred to as peaberry or caracoli. Peaberry beans are formed due to the abortion of one of the ovules in the bi-locular ovary, resulting in a single, round or rounded, seed (coffee bean) per fruit^29^. In an average coffee crop the peaberry yield ranges from 10 to 30%, but can exceed 50%^29^.

## Discussion

Using genomic SNP data analysed with STRUCTURE^28^ and principal coordinate analysis (PCoA) we confirmed the existence of hybridization between *C. liberica* and *C. dewevrei* (*C. liberica* × *C. dewevrei*). Admixture values of roughly equal proportions (e.g. 50–60%) likely represent F_1_ hybrids, while values deviating from this expectation indicate subsequent generations, including backcrosses and introgression. Clarifying the precise generational structure and direction of these hybridization events would require controlled crossing experiments and additional genomic analyses. The wide geographic distribution of admixed accessions, including samples from Central America (Costa Rica), Africa (Uganda), India, Vietnam, and South-East Asia (Peninsular Malaysia, Sarawak, Indonesia), indicates that hybridization between *C. liberica* and *C. dewevrei* is not a localised phenomenon. This pattern suggests multiple independent hybridization events, although dissemination of hybridized seed and plants across continents is also a possible scenario.

Patterns of admixture varied markedly among regions. In Sarawak, admixture proportions were biased toward *C. liberica*, whereas in India the genomic contribution of *C. dewevrei* was generally higher. These differences may reflect founder effects, i.e., what genotypes were originally sent to farms and germplasm collections in receiving countries, the relative abundance of each species in cultivation, opportunities for backcrossing, and perhaps also selective pressures associated with local environments. In the Upper Baram Valley in Sarawak, the genomic data infers establishment using a mixture of ‘pure’ *C. liberica* and admixture genotypes of *C. liberica* × *C. dewevrei*. Another scenario is that *C. liberica* × *C. dewevrei* hybrids were introduced alongside already existing farmed populations of *C. liberica*. Even though the admixture proportions in Sarawak were biased towards *C. liberica*, plants of possible F_1_ generation also exist. The origin of the seeds/plants of these coffees in the Upper Baram Valley has not been established. *Coffea liberica* was introduced to Malaysia multiple times from the 1870 s onwards^6^ and throughout the latter part of the nineteenth century^6,22^. There were also late nineteenth century introductions to many other parts of Southeast Asia^6,22^. By contrast, *C. dewevrei* was not known to science until the end of the nineteenth century, and there are no records of dissemination to Malaysia during that period or in the early part of the twentieth century. However, *C. dewevrei* was distributed widely across Africa and to Vietnam during that time^6^. It should be noted that the climate of Sarawak is more conducive to *C. liberica*, compared with *C. dewevrei*^4,22^, and thus selective environmental forces could have been acting in favour of *C. liberica.* Notably, ‘pure’ *C. dewevrei* has not been detected as a farmed plant in Peninsula Malaysia or Sarawak, via either morphological or genomic data, supporting the idea that hybrids were introduced rather than formed in situ. During the middle to latter part of the twentieth century coffee germplasm moved freely between the Americas, Africa and Asia due to institutional seed exchange programmes, and thus *C. liberica* × *C. dewevrei* or even *C. dewevrei* could have been introduced during that period. A more recent origin is also possible, perhaps within the last few decades, from a neighbouring region. The Philippines is one possible option as both species are grown there with some frequency^30^, although we also identified cultivated accession of *C. liberica* × *C. dewevrei* from Indonesia and Vietnam, providing other possible introduction origins. The likelihood of in situ hybridization in Sarawak cannot be ruled out with the data generated here. Given that *C. liberica* flowers throughout the year in Sarawak^22^, rather than over a specific period, opportunities for pollen transfer and thus outcrossing are certainly possible an even favoured. Wild hybrids between *C. liberica* and *C. dewevrei* are not a possibility, as the indigenous populations of these species are separated by at least 1000 km^4^ in Africa. In South India, admixture proportions were found to be biased towards *C. dewevrei*, possibly because this species is more frequent than *C. liberica* on farms in this part of India.

The mean values (and 1st and 3rd quartiles) for parchment thickness, seed length and seed width for *C. liberica* × *C. dewevrei* fall in-between those of the parental species (Fig. 3; Supplementary Tables S7, S8). Our preliminary analyses indicate that parchment thickness, seed length and seed width all decrease as the % admixture of *C. dewevrei* increases, and as *C. liberica* admixture % decreases (Fig. 4). Whilst the relationship between admixture percentage and these traits is not linear, it shows that introgression has a quantitative influence on these agronomic traits. Morphological observation of cultivated plants in Sarawak with high percentages of *C. dewevrei* admixture (e.g. 50–75%) showed that they had a greater number of flowers and fruits per node/axil, and a thinner fruit pulp (mostly mesocarp), compared with *C. liberica*. *Coffea liberica* usually has 4–18 flowers per node (sometimes up to 20^31^), whereas *C. dewevrei* has 6–40; the pulp (mesocarp) of *C. liberica* is usually 4–9.5 mm thick, whereas in *C. dewevrei* it is 2–3.5 mm thick^4^. The leaves of the hybrid plants may be either undisguisable from *C. liberica*, or have much larger leaves, like *C. dewevrei*. We were unable to make assumptions as to the agronomic advantage of leaf size; multilocation variety trials could help in this regard. Field observations also revealed that hybrid populations are substantially more morphologically diverse than either of the parental species, encompassing wide variation for vegetative, floral, and fruit traits. Such diversity is expected in recent hybrids and introgressed populations^32^ and contrasts with the more uniform appearance of genotypes with low levels of admixture (< 10%), which tend to resemble their predominant genomic donor species. Importantly, even low admixture genotypes may retain quantitative contributions from the minor parent, e.g. for parchment thickness, seed length and seed width (Fig. 4).

Our data and observations thus demonstrate the potential for physical trait transfer from *C. dewevrei* to *C. liberica* via hybridization. Reduced parchment thickness and thinner fruit pulp directly increase outturn by reducing non seed biomass per fruit, thereby improving the conversion of fresh fruit to clean coffee. This is particularly relevant for *C. liberica*, which is constrained by low outturns relative to other commercial coffees and *C. dewevrei*^4,6^. Reported conversion ratios for *C. liberica* (Liberica) are 8.3:1–12.5:1^33–35^; in practical terms this means that 8.3–12.5 kg of fresh fruit is required to produce 1 kg of clean coffee. This is unfavourable compared to the conversion ratios for *C. arabica* (Arabica), which has a conversion ratio of 5–6.25:1^29^, and *C. dewevrei* (excelsa), which has a outturn of 6:1–6.8:1^33,34^ or up to 8:1 and sometimes higher^6^. Improved outturn leads to increased yields per tree and per unit area, e.g. per acre or hectare. In addition to improving outturns, a thinner pulp and thinner parchment facilitate easier pulping and hulling and reduce drying times, thereby lowering labour and processing costs. If the coffee is transported in bulk as dried fruit (cherry) or parchment coffee, this would also mean reduced transport costs. Higher flower and fruit numbers per branch/node, as observed in hybrids with high *C. dewevrei* admixture, would also increase yield per tree and per unit area, compared with *C. liberica.* Collectively, these traits indicate that hybridization provides a practical route for improving the agronomic performance and economic viability of *C. liberica*.

Hybridization may also influence climatic suitability, compared with the parental species *C. dewevrei* and *C. liberica*. In Upper West Africa, *C. liberica* experiences a longer and more severe dry season with proportionally higher precipitation during the wetter/cooler months of the year, whereas much of the distribution range of *C. dewevrei* (in Central Africa) has a shorter dry season(s) with a more even annual distribution of precipitation^4^. The modelled mean annual temperature values for wild *C. liberica* and *C. dewevrei* are nearly identical, at 24.4 °C and 24.6 °C, respectively^4^. However, study of farmed *C. liberica*^22^ shows that it may be successfully cultivated over a greater range of temperature and precipitation values, compared with the species in the wild^4^. This includes a higher mean annual temperature of 25.7 °C, and a very low precipitation seasonality (Bio15^36^) which means that it has a near-consistent rainfall throughout the year. Reported Bio15 values for *C. liberica* are 45 for cultivated^22^ and 67 for wild^4^. A general, but frequent observation, is that *C. liberica* can be successfully farmed at low elevation locations (e.g. 10–100 m) and experience temperatures and precipitation values that would not be suitable for *C. dewevrei*, such as those in lowland Malaysia. Whilst the climate parameters for the successful cultivation of *C. dewevrei* have yet to be fully ascertained, it seems that this species is more suitable in slightly cooler climates with less precipitation seasonality. It is usually farmed at locations of 500–1500 m^4,6^. Thus, it is possible that *C. liberica* × *C. dewevrei* hybrids may be appropriate for cultivation under climates where either of the parents may not be suitable. Adding a proportion of *C. liberica* genome to *C. dewevrei* may enable *C. dewevrei*-like plants to survive in warmer, lower elevation locations with longer dry seasons, for example. Long-term common garden experiments for two species of poplar (*Populus fremontii* and *P. angustifolia*), the F_1_ hybrid, and backcross genotypes, showed that climate suitability traits can be transferred via hybridization in the F_1_ and in backcrossed genotypes^37^. Generally, introgressive hybridization has been important in the generation of new morphologies and adaptions to novel environments and climates compared with the parental species^32^. These factors need to be considered when identifying suitable environments for farming F_1_ hybrids and introgressed genotypes of *C. liberica* ×*C. dewevrei*, and in general when developing new, climate-suitable coffee crops. Multilocation (and multiclimate) field trials, or common garden experiments, need to be undertaken to elucidate climate suitability for *C. liberica* ×*C. dewevrei*.

Further research is required to understand pests and disease tolerance and resistance in *C. liberica* and *C. dewevrei*, and *C. liberica* × *C. dewevrei*. Tolerances and resistance could be gained, enhanced, or lost via introgression. Fungal pathogen resistance would be a key research focus, particularly for coffee leaf rust (*Hemileia vastatrix*), coffee wilt disease (*Gibberella xylarioides*), coffee berry disease (*Colletotrichum kahawae*) and various anthracnose species (*Colletotrichum* spp.). Early literature reported that *C. liberica* was not fully resistant to coffee leaf rust^23,34,38–41^, although other reports stated that the rust itself caused little harm to *C. liberica*^42,43^. The inconsistency in historical reports is likely due to, at least in part, to taxonomic and identification confusion for *C. liberica* and *C. dewevrei*. Unless the coffee species was identified with certainty, these reports must be regarded as unreliable. Observations made by us on farms in Sarawak, showed zero to negligible incidence of coffee leaf rust on *C. liberica* and *C. liberica* × *C. dewevrei*. At 12–566 m elevation, the farms in question experience high mean annual temperatures (25.7 °C), high mean annual precipitation (3633 mm), low precipitation seasonality^22^ and year-round high humidity. It has been shown that a thermal regime (TR) of 27–22 °C resulted in 2000 times higher sporulation of coffee leaf rust than with a TR of 23–18°C^44^. The germination of coffee leaf rust urediniospores and subsequent penetration into the leaf via stomata is highly dependent on leaf surface wetness^45,46^. Both of these conditions are prevalent and occur year-round in Sarawak, where *C. liberica* and *C. liberica* × *C. dewevrei* is grown^22^. *Coffea liberica* is the source of the gene SH3, which provides resistance to coffee leaf rust except for rust races with different combinations of virulence genes^47^. Indeed, *C. liberica* has been used for the breeding of coffee leaf rust resistant *C. arabica* cultivars^48^. Field observations by us indicate low to mild incidence of coffee leaf rust on *C. dewevrei* in Uganda, South Sudan, and India, but further research concerning coffee leaf rust resistance for this species is urgently required. In Sarawak we have observed a high incidence of anthracnose (*Colletotrichum* spp.) on *C. liberica* and *Coffea liberica* × *C. dewevrei*, which could be exacerbated by the warm, wet climate and high humidity^49^. Farmer and producer feedback from Uganda and South Sudan (2018–2022) report either insignificant or zero susceptibility to coffee berry disease (*Colletotrichum kahawae*) for *C. dewevrei*^6^. Coffee wilt disease (*Gibberella xylarioides*) was first reported on cultivated excelsa (*C. dewevrei*) in the Central African Republic in 1927^49^ and later caused widespread damage to farmed excelsa and robusta (*C. canephora*) across large areas of tropical Africa^50^. Coffee wilt disease is still a major constraint for robusta production but it has not been reported as a disease of excelsa during recent field surveys in Uganda and South Sudan^6^.

The coffee flavour profile of *C. dewevrei* (excelsa) is dominated by notes of dark, dried fruits (currant, prune, plum, fig), chocolate (cacao nibs, dark and milk chocolate), and spices; the coffee usually has a low to medium acidity, low bitterness, and medium to high sweetness. The sweetness is akin to Demerara sugar, maple syrup, or stevia. The coffee flavour profile of *C. liberica* (Liberica) is usually dominated by bold tropical fruit flavours, with notes of jackfruit, mango, passion fruit, longan, and various green teas; the coffee has low acidity and high sweetness. The sweetness is akin to light honey, or white sugar. *Coffea liberica* × *C. dewevrei* is rare in the global coffee value chain and currently only available in Sarawak, where it is currently sold as *C. liberica.* The flavour of this coffee, as sampled from Sarawak, combines features of the parental species, with perhaps a more accessible flavour profile and an improved balance of fruit and other flavour notes, compared with *C. liberica*. Our preliminary evaluations indicate that *Coffea liberica* × *C. dewevrei* hybrids could be of value for flavour, quality and diversity enhancements for both *C. liberica* and *C. dewevrei*. Rigorous sensory evaluation across multiple environments and processing methods will be required to substantiate these initial impressions.

A key observation from this study is the negligible (< 5%) to low incidence (10–20%) of single-seeded fruits with rounded seeds, often known as peaberry or caracoli^29,51^. A high proportion of peaberry (50% or more) is usually linked to genetic factors, possibly including pollen-stylar incompatibility^52^ and pairing complications during meiosis. This is a common feature in interspecies and intraspecies F1 and F2 coffee hybrids^51^. There may be other causes for the formation of peaberry, aside from genetic issues, such as poor plant health and unsuitable weather and climate conditions^29^. The low incidence in *C. liberica* × *C. dewevrei* indicates genomic compatibility, probably due to the close evolutionary relationship between the two species^4^. A high proportion (50%, or more) of peaberry, as detected in some interspecies *Coffea* hybrids is considered as a major constraint in coffee breeding, especially when associated with low yields. High yields in interspecies coffee hybrids, suggest genomic compatibility, as found in the interspecies hybrid *C. canephora* × *C. congensis*^13,53^. *Coffea liberica* × *C. dewevrei* and *C. canephora* × *C. congensis* hybrids are the result of crossing between closely related species^4,54,55^. Reproductive compatibility, and the resulting low proportion of peaberry formation means that *C. liberica* × *C. dewevrei* hybrids could be readily deployed as crop plants.

Improved genotypes resulting from *C. liberica* × *C. dewevrei* hybrids could be brought into production relatively quickly by clonal propagation, via cuttings, micropropagation (in vitro), or by grafting on to selected rootstocks, such as *C. liberica* and *C. dewevrei*. Pure breeding lines are groups of individuals with reduced genetic variation that produce offspring via seed, with consistent traits representing uniform phenotypes. This might be an option for *C. liberica* × *C. dewevrei*, although it is unknown how many generations it would take to achieve a workable level of variation to produce a pure line cultivar derived from *C. liberica* × *C. dewevrei*.

To facilitate communication and recognition of this interspecies hybrid, we propose the formal name *C.* × *libex* for hybrids between *C. liberica* and *C. dewevrei*. This follows established conventions for naming interspecies hybrids^56^, such as the interspecies hybrid *C. canephora* × *C. congensis* which is formally named as *C*. × *crameri* and also referred to as ‘Congusta’. Cultivars arising from *C.* × *libex* could be designated by using a cultivar name placed in single quotes^56^, as in the example of *Coffea* ‘Congusta’. For the methodology employed here, we suggest an admixture cut-off of 10% for the recognition of *C. liberica* × *C. dewevrei* (*C.* × *libex*). For some of our data, and when using other genomic methods, such a small proportion of genomic admixture might represent noise but in some cases it could represent the transfer of meaningful genetic material and thus identifiable and useful traits.

## Methods

### DNA sampling and sequencing

We sampled 113 accessions of *C. liberica*, *C. dewevrei*, and putative hybrids, sourced primarily from cultivated material, including farms and germplasm collections. Wild sourced accessions were included as reference standards. Of these, seven accessions of *C. liberica* and 20 of *C. dewevrei* had been unambiguously identified to species in a previous genomic morphological study, using a comprehensive sampling of related *Coffea* species^4^. Owing to the high frequency of suspected hybrids more rigorous sampling was undertaken in Sarawak. Sampling details, other accession information and sequence information are provided in Supplementary Table S1. Accepted botanical names and authorities follow the International Plant Names Index (https://www.ipni.org).

Total DNA was extracted from herbarium leaf tissue, silica gel dried leaves, or seeds using a modified CTAB protocol for herbarium specimens^57^. Sequence target capture data were generated using the universal Angiosperms353 target capture kit developed to retrieve 353 nuclear genes across the angiosperms^24,25^. The Angiosperms353 target capture kit, is a genomic tool that has been used to resolve relationships at various levels of taxonomic hierarchy in flowering plants^26,27,58^, including at the population scale^59–61^.

Genomic libraries were constructed using an optimized protocol^62^ for half volumes of the NEBNext Ultra II DNA Library Prep kit for Illumina (New England Biolabs) and purified using AMPure XP magnetic beads and multiplexed using NEBNext Multiplex Oligos for Illumina (Dual Index Primer Sets I and II). Pools containing 113 genomic libraries mixed in equimolar conditions were enriched with half reactions of the Angiosperms353 probe kit following the myBaits kit manual v.3.02 (Arbor Biosciences), using an optimized protocol^63^. The DNA concentration and fragment size distribution were calculated using a Quantus fluorometer (Promega Corp.) and an Agilent 4200 TapeStation (Agilent Technologies), respectively. Sequencing was performed on a HiSeq (Illumina Inc.) by Macrogen, producing 2 × 150 bp paired-end reads. Raw reads were submitted to the European Nucleotide Archive (https://www.ebi.ac.uk). ID codes are given in Supplementary Table S6.

### SNP calling production and genomic analyses

Trimmomatic v.0.35^64^ was used to discard low-quality reads and trim adaptors based on the reports generated by FastQC v.0.11.7^65^ and HybPiper v.2.3.0^66^ to retrieve the 353 nuclear loci using a combination of map to reference and de novo assembly methods for all samples.

The 113 accessions were divided into three groups, (1) *C. liberica* and (2) *C. dewevrei*, based on key morpho-taxonomic characters^4,6^, and (3) suspected hybrids between these two species. To generate SNP data for these three groups, we used the framework developed by DePristo et al.^67^using GATK^68^ following the pipelines established for Angiosperms353 data^27,59^. This process combined aligned and unaligned reads to a reference built with the longest exon obtained for all samples. We removed duplicate sequences and performed joint genotype calling for all samples after initially generating variants for each sample individually^53^ in a variant call format (VCF) file. The initial VCF file was processed with a stringent filter (QD < 5.0 || FS > 60.0 || MQ < 40.0 || MQRankSum < − 12.5 || ReadPosRankSum < − 8.0), removing indels and SNPs with missing data using GATK and eliminating linked SNPs with PLINK^69^. Base quality score recalibration was performed in GATK, followed by a repeated variant calling step.

To exclude the likelihood of genome admixture (via hybridization) from other coffee species, we first ran all 113 accessions against a Liberica alliance SNP dataset^4^, and an unpublished data set of multiple *Coffea* species, which showed that all accessions aligned with either *C. liberica* or *C. dewevrei* (results not shown).

To examine genetic differentiation patterns for the accession of *C. liberica*, *C. dewevrei*, and potential hybrids we used STRUCTURE^28^ to determine genetic clusters in the dataset. The online tool, StructureSelector^70^ was used to calculate the most likely value of *K* for various estimators, including Δ*K (REF).* A *K* value of *K* = 2 was manually set based on the optimal recovered *K* value and the number of species; *K* = 3 and *K* = 4 were also examined. Each *K* was analysed with ten replicates, using 100,000 burn-in iterations followed by 1,000,000 Markov chain Monte Carlo repetitions. The most likely number of clusters was identified using PLINK outputs and were converted into STRUCTURE-compatible files using PGDSpider^71^. The results from STRUCTURE were visualized with StructRly^72^(Fig. 1b, Supplementary Fig. S1). Genetic differentiation was explored using a genetic distance-based phylogenetic tree (Fig. 1a) using upgma with poppr 2.9.6, adegenet, ape 5.7.1 and MetBrewer^73^(Fig. 2) and PCoA in R 4.3.0^74^, with the adegenet and ggplot2 packages, retaining three principal components to investigate genetic groupings (Fig. 1c).

### Morphological analysis of seed and parchment

Parchment (endocarp) thickness and seed size values provide useful characters for the differentiation of *C. liberica* and *C. dewevrei*^4^. Reported values for parchment thickness are: *C. liberica* 0.36–0.77 mm (mean value 0.57 mm) and *C. dewevrei* 0.22–0.41 mm (mean value 0.31 mm). Seed length and width for *C. liberica* is 9.5–18.3 and 6.5–12 mm, respectively (mean values 12.6 × 8.4 mm) and *C. dewevrei* 7.7–11.3 and 5.4–8 mm, respectively (mean values 9.3 × 6.6 mm)^4^. We measured the parchment thickness of 477 samples (29 for *C. liberica*, 244 for *C. dewevrei*, and 204 for the putative hybrid accessions); accession details are provided in Supplementary Table S8. We measured the seed length and seed width of 2,140 seeds (467 for *C. liberica*, 781 for *C. dewevrei*, and 892 for the hybrid accessions); accession details are given in Supplementary Table S8. Measurements of parchment thickness was made using a Mitutoyo 193 − 111 0–25 mm (0.001 mm) micrometer, and for seed length and width a 0–150 mm (0.01 mm) Mitutoyo 500-196-30 digital Vernier calliper. One way ANOVA was used to examine the variance for each of the three characters, followed by a TukeyHSD test to identify if the means for specific pairs, of the three study groups, were significantly different. Box plots for parchment thickness, seed length, and seed width are given in Fig. 3; a scatter plot of seed length vs. seed width is given in Supplementary Fig. S2. Descriptive statistics and the results of the statistical tests are given in Supplementary Tables S5. Parchment thickness, seed length and seed width were plotted against admixture % (Fig. 4) to determine the relationships between these physical traits and admixture.

## Supplementary Information

Below is the link to the electronic supplementary material.

Supplementary Material 1

## Data Availability

Raw reads for Angiosperms353 sequence data are available at the European Nucleotide Archive ([https://www.ebi.ac.uk](https:/www.ebi.ac.uk)) under project no. [to update, post review]; ID codes are given in Supplementary Table S6.
