# Supplementary material for: Genomic elucidation of hybridization between Liberica and excelsa coffee and its implications for coffee crop development
Source: Sci Rep. 2026 May 14;16:22107. doi: 10.1038/s41598-026-49305-5 (PMC13369886; doi:10.1038/s41598-026-49305-5)
Supplement: Supplementary file 1 — Supplementary Material 1 [file 41598_2026_49305_MOESM1_ESM.pdf]

Supplementary Information

FIGURES

Supplementary Fig. S1. Alternative STRUCTURE K values for *C. liberica*, *C. dewevrei* and *C. liberica* × *C. dewevrei*

Supplementary Fig. S2. Seed length versus seed width for C. liberica, C. dewevrei and *C. liberica* × *C. dewevrei*

TABLES

Supplementary Table S1. List of DNA samples with key accession data and sequencing details

Supplementary Table S2. Admixture percentages for K = 2

Supplementary Table S3. Admixture percentages for K = 3 and K = 4

Supplementary Table S4. Admixture percentages K = 2 for Malaysian accessions

Supplementary Table S5. Descriptive statistics and details of ANOVA and TukeyHSD test: parchment thickness, seed length and width

Supplementary Table S6. European Nucleotide Archive ID and file codes

Supplementary Fig. S1. Alternative STRUCTURE K values for *C. liberica*, *C. dewevrei* and *C. liberica* × *C. dewevrei*. Analyses based on 7,618 exon region SNPs for 107 samples. Group 1 = *C. dewevrei*; group 2 = *C. liberica*; group 3 = *C. liberica* × *C. dewevrei*. *K* values of *K* = 3 and *K* = 4.

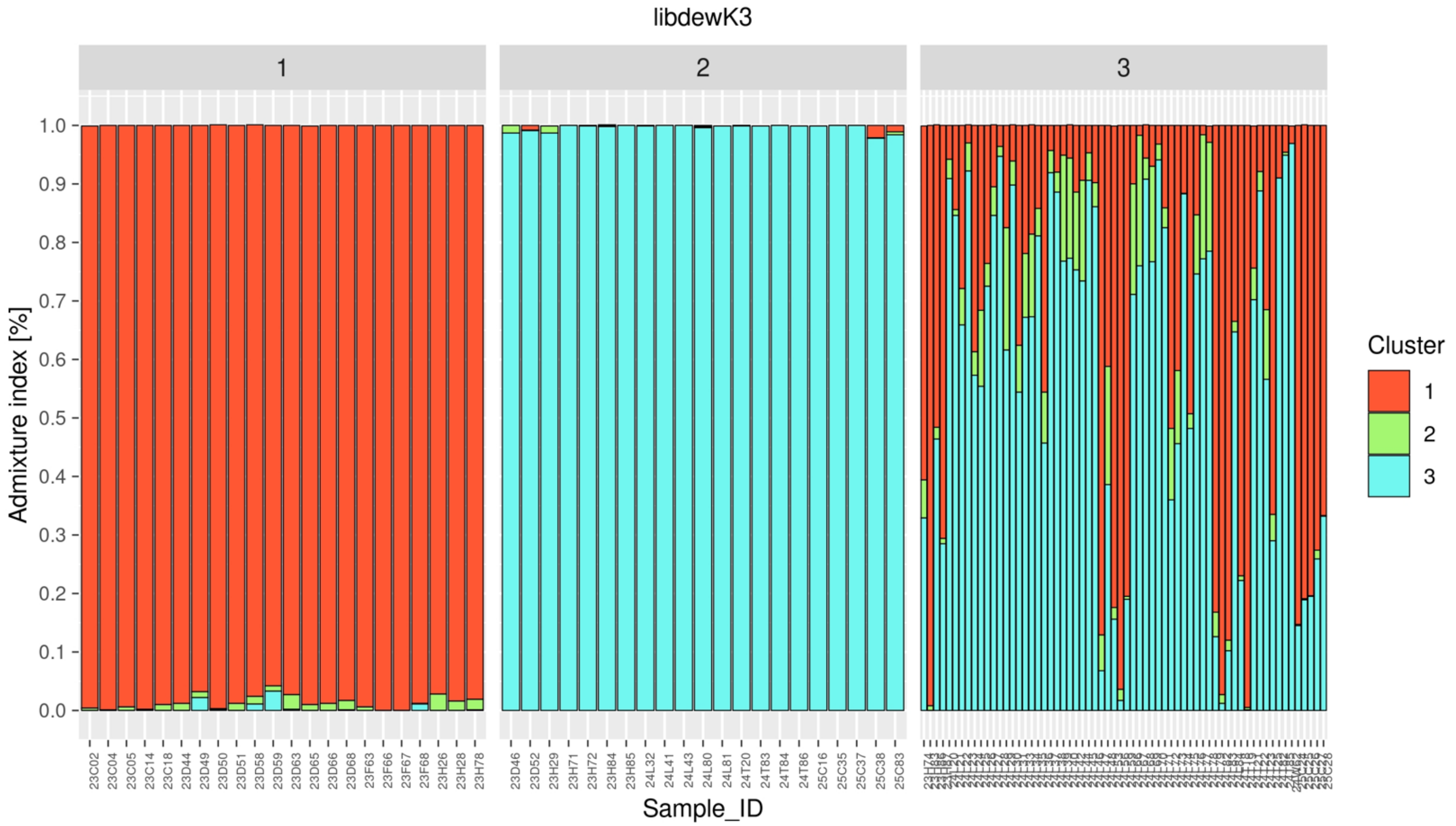

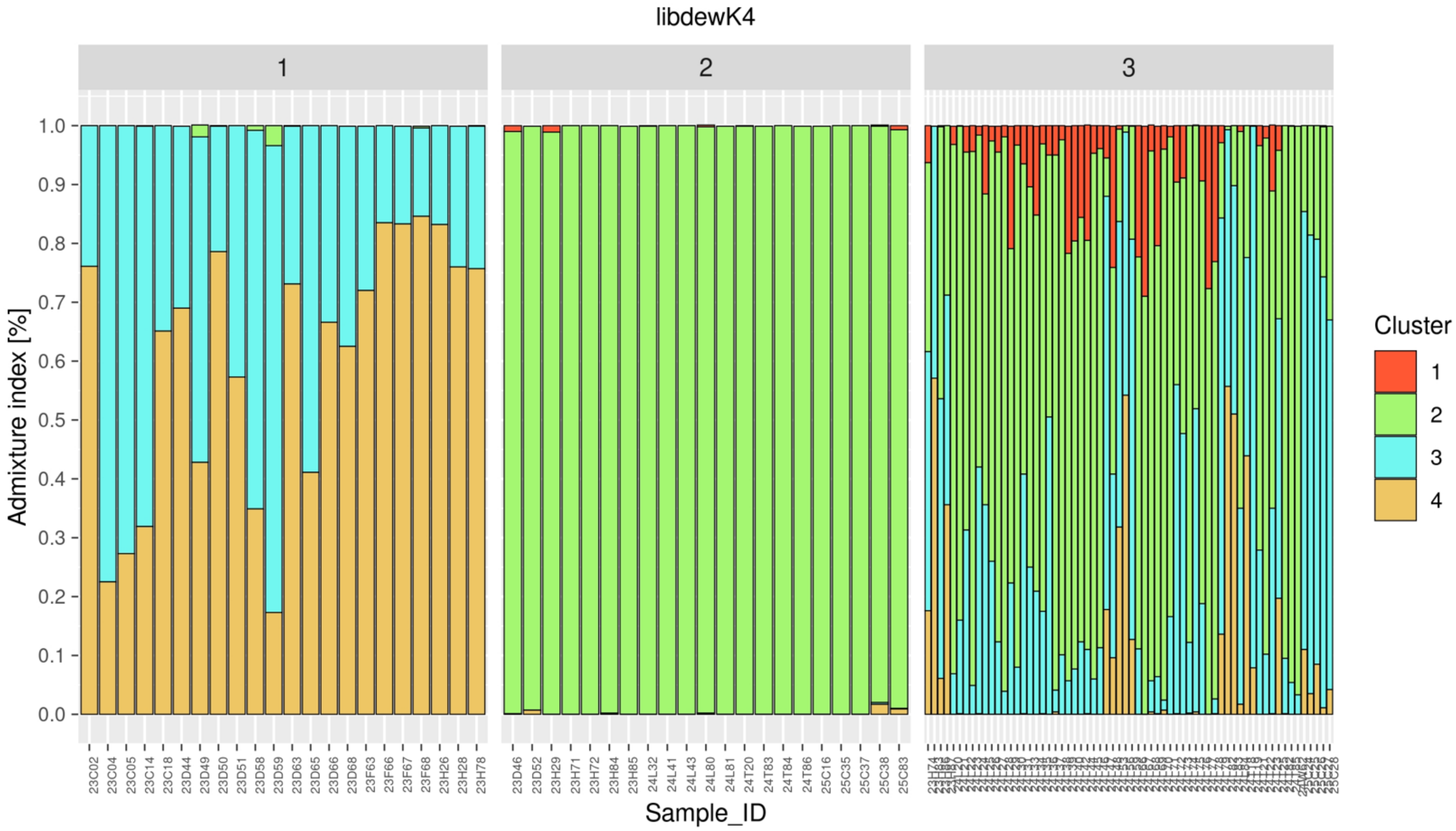
libdewK4
1
2
3
Admixture index [%]
1.0
0.9
0.8
0.7
0.6
0.5
0.4
0.3
0.2
0.1
0.0
23C02 23C04 23C05 23C14 23C18 23D44 23D49 23D50 23D51 23D58 23D59 23D63 23D65 23D66 23D68 23F63 23F66 23F67 23F68 23H26 23H28 23H78
23D46 23D52 23H29 23H71 23H72 23H84 23H85 24L32 24L41 24L43 24L80 24L81 24T20 24T83 24T84 24T86 25C16 25C35 25C37 25C38 25C83
Sample_ID
Cluster
1
2
3
4

Supplementary Fig. S2. Seed length versus seed width for C. liberica, C. dewevrei and *C. liberica* × *C. dewevrei*. This scatter plot includes the measurement of 2,140 seeds (unroasted coffee) beans). Commercial samples are unscreened (i.e. not size-sorted by the producer). See Supplementary Table S8 for accession information.

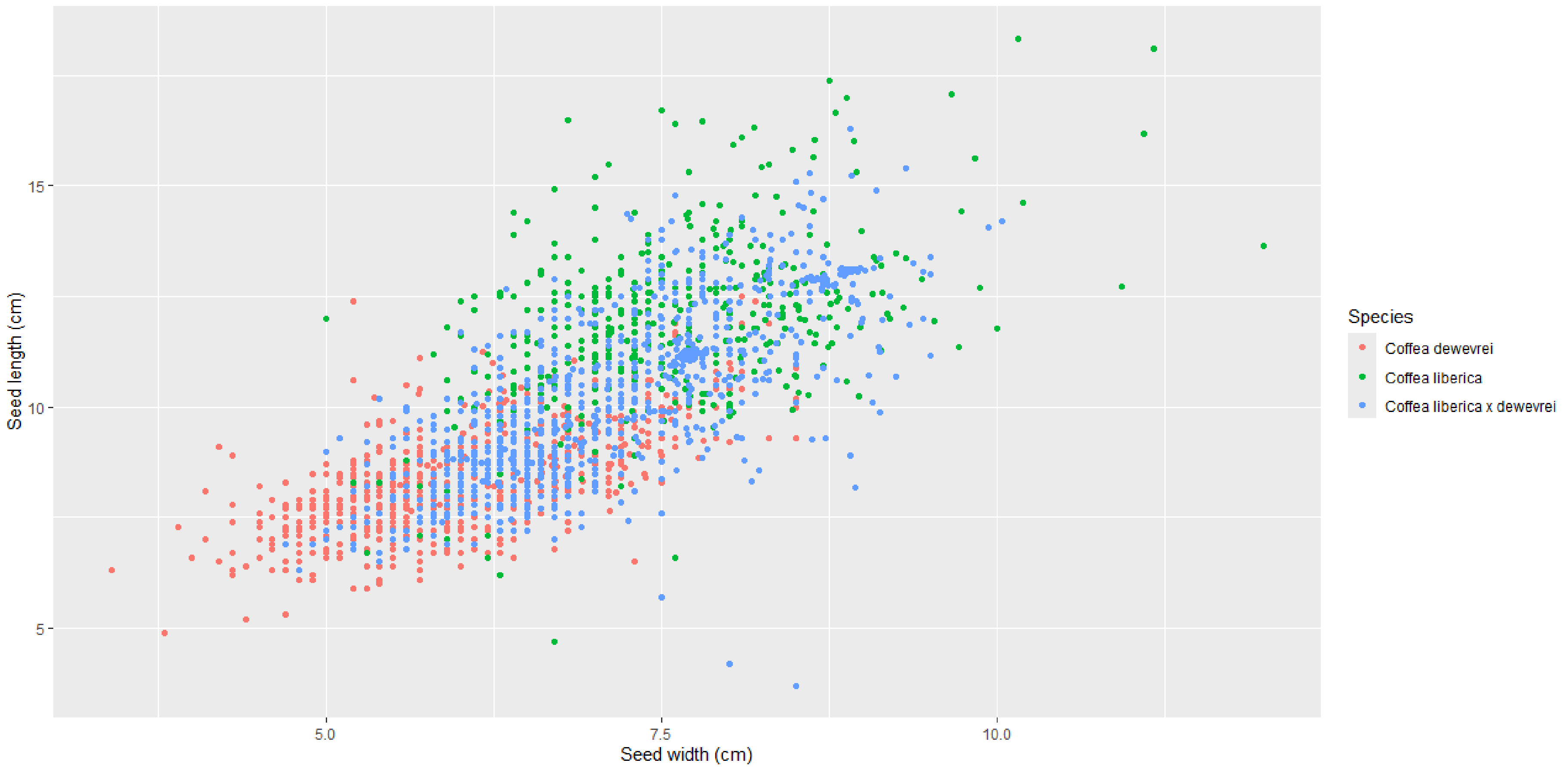

Supplementary Table S1. List of DNA samples with key accession data and sequencing details.

Voucher specimens held at the herbarium, Royal Botanic Gardens, Kew (K).

Taxon	Country	Year of collection	Collector name and number	Material type	Cult or wild	DNA Library code	No. of quality-filtered paired reads	No. of reads on target	Enrichment efficiency (% reads on target)	No. of genes assembled at 50% [for 353 genes]	Total bp recovered [for 353 genes]	Efficiency recovery (% bp recovered compared to reference)
Coffea dewevrei	CAR	2003	Sonke 3096	Leaf (herb. K)	wild	23C14	875,954	460,122	52.0	280	208,305	72.4
Coffea dewevrei	CAR	1982	Fay 5187	Leaf (herb. K)	wild	23C18	4,437,896	2,974,267	66.1	319	245,724	85.4
Coffea dewevrei	CAR	2001	IRD EB53	Leaf (silica)	wild	23D66	5,329,126	2,258,127	41.9	331	259,059	90.1
Coffea dewevrei	CAR	2001	IRD EB60	Leaf (silica)	wild	23D68	8,731,736	3,977,756	45.0	328	259,155	90.1
Coffea dewevrei	Costa Rica	2019	Hernandez/Coffea diversa 3692	Seed (comm.)	cult.	23D49	3,453,136	1,846,425	52.8	320	247,506	86.0
Coffea dewevrei	Costa Rica	2019	Hernandez/Coffea diversa 3716	Seed (comm.)	cult.	23H83	5,222,832	2,912,709	55.0	333	261,132	90.8
Coffea dewevrei	Costa Rica	2019	Hernandez/Coffea diversa 3691	Seed (comm.)	cult.	23D56	2,784,546	1,291,555	45.8	328	256,878	89.3
Coffea dewevrei	Costa Rica	2019	Hernandez/Coffea diversa 3485	Seed (comm.)	cult.	23H75	7,010,712	3,722,832	52.4	332	261,393	90.9
Coffea dewevrei	Costa Rica	2019	Hernandez/Coffea diversa 3475	Seed (comm.)	cult.	23D53	3,461,430	1,456,824	41.6	326	256,203	89.1
Coffea dewevrei	Costa Rica	2019	Hernandez/Coffea diversa 3559	Seed (comm.)	cult.	23D57	2,550,210	1,082,776	42.0	321	252,471	87.8
Coffea dewevrei	Costa Rica	2019	Hernandez/Coffea diversa 3694	Seed (comm.)	cult.	23C01	3,439,840	1,931,062	55.3	328	257,622	89.6
Coffea dewevrei	Costa Rica	2019	Hernandez/Coffea diversa 3449	Seed (comm.)	cult.	23D54	3,821,088	1,771,340	50.0	324	254,337	88.4
Coffea dewevrei	DRC	2002	BR 2174	Leaf (silica)	wild	23D44	4,699,966	2,288,194	48.1	328	258,123	89.7

Coffea dewevrei	DRC	2002	Billiet F BR 19370062	Leaf (silica)	wild	23D65	6,422,782	3,086,845	47.4	329	257,184	89.4
Coffea dewevrei	DRC	1996	Liengola ILB130	Leaf (herb)	wild	23H78	2,538,432	1,307,975	51.2	304	233,463	81.2
Coffea dewevrei	Guinea	2015	Couch 757	Leaf (herb. K)	cult. (farm)	23D51	4,386,296	2,170,879	48.9	324	257,190	89.4
Coffea dewevrei	Hawaii	2019	Kawabata A9	Seed (comm.)	cult.	23D58	3,033,600	1,329,352	43.3	324	254,562	88.5
Coffea dewevrei	Hawaii	2019	Kawabata A10	Seed (comm.)	cult.	23D59	2,454,936	1,103,466	44.5	323	251,331	87.4
Coffea dewevrei	India	2024	PBG 155	Leaf (dried)	cult.	24L82	7,707,182	1,407,484	18.4	320	244,227	84.9
Coffea dewevrei	India	2024	Dashrath 34	Leaf (dried)	cult. (farm)	24L56	8,775,570	1,938,552	22.0	323	252,216	87.7
Coffea dewevrei	Sierra Leone	2019	Sarmu SL 4	Seed (comm.)	cult. (farm)	23D50	3,656,938	1,773,361	47.9	322	251,262	87.4
Coffea dewevrei	Sierra Leone	2019	Sarmu SL 3	Seed (comm.)	cult. (farm)	23C02	3,568,366	2,229,106	61.5	327	258,090	89.7
Coffea dewevrei	Sierra Leone	2013	CEPAH	Leaf (dried)	cult.	23F63	3,186,524	1,192,457	36.9	311	238,062	82.8
Coffea dewevrei	Sierra Leone	2013	Haggar 14-16	Leaf (silica)	cult.	23F66	4,357,284	2,272,340	51.4	333	260,361	90.5
Coffea dewevrei	Sierra Leone	2017	Haggar 16-16	Leaf (silica)	cult.	23F67	4,599,306	2,618,562	56.0	335	262,158	91.1
Coffea dewevrei	Sierra Leone	2017	Haggar 18-16	Leaf (silica)	cult.	23F68	5,893,936	2,997,985	50.3	325	253,644	88.2
Coffea dewevrei	South Sudan	1930	Snowden 1697	Leaf (herb. K)	wild	23D63	7,496,486	3,767,361	49.8	318	248,862	86.5
Coffea dewevrei	South Sudan	1980	Friis & Vollesen 1981	Leaf (herb. K)	wild	23H28	5,387,598	2,044,596	37.7	320	241,479	84.0
Coffea dewevrei	Uganda	2016	Slow Foods	Seed (comm.)	cult.	23C04	3,889,896	2,404,736	60.9	328	256,209	89.1
Coffea dewevrei	Uganda	2016	Slow Foods	Seed (comm.)	cult.	23C05	4,463,406	2,726,510	60.1	329	258,393	89.8
Coffea dewevrei	Uganda	1941	Thomas 4026	Seed (herb. K)	wild	23H26	5,124,836	2,677,659	51.6	332	262,125	91.1

Coffea dewevrei	Vietnam	2023	Lee Wee Ting EC 007	Leaf (dried)	cult. (farm)	24T19	3,991,722	2,082,267	52.3	334	256,632	89.2
**Taxon**	**Country**	**Year of collection**	**Collector name and number**	**Material type**	**Cult. (C) or wild (W)**	**DNA Library code**	**No. of quality-filtered paired reads**	**No. of reads on target**	**Enrichment efficiency (% reads on target)**	**No. of genes assembled at 50% [[for 353 genes]**	**Total bp recovered [for 353 genes]**	**Efficiency recovery (% bp recovered compared to reference)**
Coffea liberica	Ivory Coast	2002	IRD EA 67	Leaf (silica)	wild	23D46	4,577,748	2,479,424	53.4	329	259,539	90.2
Coffea liberica	Cameroon	1918	Aunet 315	Leaf (herb. P)	cult.	25C83	4,908,534	1,460,330	30.0	296	214,059	74.4
Coffea liberica	Dominica	1884	Nicholls [EBC 53497]	Seed (EBC)	cult.	25C38	5,625,920	1,608,279	29.0	309	224,271	78.0
Coffea liberica	India	2024	H1	Leaf (dried)	cult.	25C16	6,979,650	3,590,866	51.6	335	256,929	89.3
Coffea liberica	Madagascar	2023	Davis L2	Leaf (dried)	cult. (farm)	23H71	4,102,946	1,366,550	33.0	333	258,642	89.9
Coffea liberica	Madagascar	2023	Davis L3.5	Leaf (dried)	cult. (farm)	23H72	3,450,548	1,442,817	41.4	330	258,306	89.8
Coffea liberica	Nigeria	1895	Millen [EBC 53476]	Seed (EBC)	cult./wild	23H85	3,848,296	2,426,939	62.5	318	239,517	83.3
Coffea liberica	Peninsular Malaysia	2020	House of Kendal A125	Seed (comm.)	cult. (farm)	23D52	3,397,834	1,698,992	49.5	327	254,184	88.4
Coffea liberica	Peninsular Malaysia	2023	Lee Wee Ting EC 004	Leaf (dried)	cult. (farm)	24L80	2,955,240	514,810	17.5	314	236,412	82.2
Coffea liberica	Peninsular Malaysia	1896	Govt. of Perak. [EBC 53496]	Seed (EBC)	cult.	25C35	9,978,916	3,535,338	35.6	328	245,265	85.3
Coffea liberica	Peninsular Malaysia	2024	Lee Wee Ting EC 017	Leaf (dried)	cult. (farm)	24T86	4,753,800	985,581	21.7	331	250,845	87.2
Coffea liberica	Peninsular Malaysia	2024	Lee Wee Ting EC 013	Leaf (dried)	cult. (farm)	24T82	3,069,888	958,891	31.7	328	253,992	88.3
Coffea liberica	Peninsular Malaysia	2024	Lee Wee Ting EC 015	Leaf (dried)	cult. (farm)	24T84	7,831,782	2,026,008	26.1	332	254,352	88.4
Coffea liberica	Sarawak	2023	Lee Wee Ting LBC 014	Leaf (dried)	cult. (farm)	24L32	5,141,826	2,762,228	52.9	331	257,256	89.4

Coffea liberica	Sarawak	2023	Lee Wee Ting LBC 023	Leaf (dried)	cult. (farm)	24L41	8,447,298	3,922,821	45.9	331	259,503	90.2
Coffea liberica	Sarawak	2023	Lee Wee Ting LBC 025	Leaf (dried)	cult. (farm)	24L43	9,977,578	4,546,104	45.0	326	255,888	89.0
Coffea liberica	Sarawak	2023	Lee Wee Ting EC 005	Leaf (dried)	cult. (farm)	24L81	6,975,162	1,212,231	17.4	320	242,166	84.2
Coffea liberica	Sarawak	2023	Lee Wee Ting EC 009	Leaf (dried)	cult. (farm)	24T20	4,228,018	2,181,094	52.1	339	258,234	89.8
Coffea liberica	Sierra Leone	2013	Swaray 213	Leaf (herb. K)	wild	23H29	8,752,626	3,910,987	44.2	331	252,585	87.8
Coffea liberica	Sierra Leone	1924	unknown [EBC 53477]	Seed (EBC)	cult.	25C37	6,755,100	2,211,999	33.0	329	247,662	86.1
Coffea liberica	Sri Lanka	1892	Dr Trimen [EBC 53740]	Seed (EBC)	cult.	23H84	5,038,434	3,382,357	66.2	326	247,659	86.1
**Taxon**	**Country**	**Year of collection**	**Collector name and number**	**Material type**	**Cult. (C) or wild (W)**	**DNA Library code**	**No. of quality-filtered paired reads**	**No. of reads on target**	**Enrichment efficiency (% reads on target)**	**No. of genes assembled at 50% [[for 353 genes]**	**Total bp recovered [for 353 genes]**	**Efficiency recovery (% bp recovered compared to reference)**
Coffea liberica × *C. dewevrei*	Indonesia	2023	Lee Wee Ting EC 002	Seed (comm.)	cult. (farm)	24L48	8,861,486	4,665,343	51.9	335	260,583	90.6
Coffea liberica × *C. dewevrei*	Indonesia	2023	Lee Wee Ting EC 003	Leaf (dried)	cult. (farm)	24L79	4,991,584	823,521	16.5	324	248,079	86.2
Coffea liberica × *C. dewevrei*	India	2024	BMMK2	Leaf (dried)	cult.	25C24	9,886,770	3,157,361	32.0	330	253,929	88.3
Coffea liberica × *C. dewevrei*	India	2024	BMMK3	Leaf (dried)	cult.	25C25	6,752,242	1,522,565	22.6	323	244,290	84.9
Coffea liberica × *C. dewevrei*	India	2024	BMMK4	Leaf (dried)	cult.	25C26	6,502,722	2,959,338	45.6	329	249,975	86.9
Coffea liberica × *C. dewevrei*	India	2024	BMMK5	Leaf (dried)	cult.	25C27	5,003,084	1,431,349	28.7	326	244,359	85.0
Coffea liberica × *C. dewevrei*	India	2024	BMMK6	Leaf (dried)	cult.	25C28	8,252,248	1,875,069	22.8	322	242,085	84.2
Coffea liberica × *C. dewevrei*	India	2024	Mysore 9	Leaf (dried)	cult.	24L59	6,059,464	3,016,916	49.1	331	259,182	90.1

Coffea liberica × *C. dewevrei*	India	2024	Dashrath 32	Leaf (dried)	cult. (farm)	24L53	6,379,468	2,237,952	34.8	328	253,542	88.1
Coffea liberica × *C. dewevrei*	India	2024	PBG 154	Leaf (dried)	cult.	24L83	4,716,410	827,053	17.6	322	242,691	84.4
Coffea liberica × *C. dewevrei*	Costa Rica	2019	Hernandez/Coffea Diversa 3476	Seed (comm.)	cult.	23H74	6,365,100	3,721,406	57.6	332	259,956	90.4
Coffea liberica × *C. dewevrei*	Uganda	1924	unknown [EBC 53449]	Seed (EBC)	cult.	23H86	5,027,152	3,172,368	62.2	325	249,933	86.9
Coffea liberica × *C. dewevrei*	Costa Rica	2019	Hernandez/Coffea Diversa 3450	Seed (comm.)	cult.	23H87	7,618,466	3,913,878	50.9	329	260,226	90.5
Coffea liberica × *C. dewevrei*	Sarawak	2023	Lee Wee Ting LBC 002	Leaf (dried)	cult. (farm)	24L21	4,060,872	1,371,937	33.5	317	237,705	82.6
Coffea liberica × *C. dewevrei*	Sarawak	2023	Lee Wee Ting LBC 003	Leaf (dried)	cult. (farm)	24L22	5,082,692	1,661,902	32.4	324	245,136	85.2
Coffea liberica × *C. dewevrei*	Sarawak	2023	Lee Wee Ting LBC 005	Leaf (dried)	cult. (farm)	24L24	5,507,172	1,641,028	29.6	322	244,656	85.1
Coffea liberica × *C. dewevrei*	Sarawak	2023	Lee Wee Ting LBC 007	Leaf (dried)	cult. (farm)	24L25	10,022,734	5,601,025	55.0	328	254,493	88.5
Coffea liberica × *C. dewevrei*	Sarawak	2023	Lee Wee Ting LBC 008	Leaf (dried)	cult. (farm)	24L26	7,808,724	4,344,729	54.8	331	256,827	89.3
Coffea liberica × *C. dewevrei*	Sarawak	2023	Lee Wee Ting LBC 009	Leaf (dried)	cult. (farm)	24L27	5,166,564	2,771,836	52.8	332	259,911	90.4
Coffea liberica × *C. dewevrei*	Sarawak	2023	Lee Wee Ting LBC 011	Leaf (dried)	cult. (farm)	24L29	10,860,150	6,260,794	56.7	329	257,811	89.6
Coffea liberica × *C. dewevrei*	Sarawak	2023	Lee Wee Ting LBC 013	Leaf (dried)	cult. (farm)	24L31	8,087,032	4,261,435	51.9	331	257,139	89.4
Coffea liberica × *C. dewevrei*	Sarawak	2023	Lee Wee Ting LBC 015	Leaf (dried)	cult. (farm)	24L33	10,900,260	6,403,588	57.8	329	254,490	88.5
Coffea liberica × *C. dewevrei*	Sarawak	2023	Lee Wee Ting LBC 016	Leaf (dried)	cult. (farm)	24L34	7,057,756	4,172,062	58.1	332	256,794	89.3
Coffea liberica × *C. dewevrei*	Sarawak	2023	Lee Wee Ting LBC 017	Leaf (dried)	cult. (farm)	24L35	9,272,228	5,424,189	57.5	332	257,457	89.5
Coffea liberica × *C. dewevrei*	Sarawak	2023	Lee Wee Ting LBC 018	Leaf (dried)	cult. (farm)	24L36	4,504,116	2,402,913	52.6	332	255,933	89.0
Coffea liberica × *C. dewevrei*	Sarawak	2023	Lee Wee Ting LBC 020	Leaf (dried)	cult. (farm)	24L38	6,486,596	3,714,405	56.3	328	253,095	88.0

Coffea liberica × *C. dewevrei*	Sarawak	2023	Lee Wee Ting LBC 021	Leaf (dried)	cult. (farm)	24L39	5,261,288	2,093,718	39.4	327	253,674	88.2
Coffea liberica × *C. dewevrei*	Sarawak	2023	Lee Wee Ting LBC 022	Leaf (dried)	cult. (farm)	24L40	2,851,710	1,489,801	51.6	325	254,007	88.3
Coffea liberica × *C. dewevrei*	Sarawak	2023	Lee Wee Ting LBC 024	Leaf (dried)	cult. (farm)	24L42	6,548,350	3,649,568	54.9	333	258,075	89.7
Coffea liberica × *C. dewevrei*	Sarawak	2023	Lee Wee Ting LBC 026	Leaf (dried)	cult. (farm)	24L44	11,103,878	5,352,154	47.6	327	255,279	88.8
Coffea liberica × *C. dewevrei*	Sarawak	2023	Lee Wee Ting LBC 028	Leaf (dried)	cult. (farm)	24L46	7,979,262	3,457,367	42.8	325	252,888	87.9
Coffea liberica × *C. dewevrei*	Sarawak	2023	SLG 1	Leaf (dried)	cult. (farm)	24L66	9,598,354	2,850,992	29.6	330	255,813	88.9
Coffea liberica × *C. dewevrei*	Sarawak	2023	SLG 2	Leaf (dried)	cult. (farm)	24L67	8,491,728	3,293,649	38.4	329	255,714	88.9
Coffea liberica × *C. dewevrei*	Sarawak	2023	SLG 4	Leaf (dried)	cult. (farm)	24L69	5,926,918	2,038,863	34.1	329	257,295	89.5
Coffea liberica × *C. dewevrei*	Sarawak	2023	SLG 6	Leaf (dried)	cult. (farm)	24L71	9,272,650	3,899,496	41.6	309	238,974	83.1
Coffea liberica × *C. dewevrei*	Sarawak	2023	SLG 7	Leaf (dried)	cult. (farm)	24L72	4,086,664	1,283,393	31.2	325	255,846	88.9
Coffea liberica × *C. dewevrei*	Sarawak	2024	SLG 8	Leaf (dried)	cult. (farm)	24L73	4,895,700	656,090	13.4	315	240,405	83.6
Coffea liberica × *C. dewevrei*	Sarawak	2024	Lee Wee Ting LBC 031	Leaf (dried)	cult. (farm)	24L74	5,357,920	759,275	14.2	320	243,132	84.5
Coffea liberica × *C. dewevrei*	Sarawak	2024	Lee Wee Ting LBC 030	Leaf (dried)	cult. (farm)	24L75	3,911,844	542,382	13.9	323	243,564	84.7
Coffea liberica × *C. dewevrei*	Sarawak	2024	Lee Wee Ting LBC 029	Leaf (dried)	cult. (farm)	24L76	9,397,316	1,579,663	16.9	326	249,063	86.6
Coffea liberica × *C. dewevrei*	Sarawak	2023	SLG 9	Leaf (dried)	cult. (farm)	24L77	5,295,224	765,610	14.5	317	242,658	84.4
Coffea liberica × *C. dewevrei*	Indonesia	2023	Lee Wee Ting EC 002	Leaf (dried)	cult. (farm)	24L78	11,132,634	1,841,861	16.6	321	246,924	85.8
Coffea liberica × *C. dewevrei*	Costa Rica	2019	Hernandez/ Coffea Diversa 3419	Seed (comm.)	cult.	24T18	2,894,664	1,413,119	48.9	330	254,061	88.3
Coffea liberica × *C. dewevrei*	Sarawak	2023	Lee Wee Ting EC 010	Leaf (dried)	cult. (farm)	24T21	5,003,478	2,565,778	51.8	332	256,092	89.0

Coffea liberica × *C. dewevrei*	Sarawak	2023	Lee Wee Ting EC 011	Leaf (dried)	cult. (farm)	24T22	3,720,874	1,990,668	53.8	335	255,936	89.0
Coffea liberica × *C. dewevrei*	Sarawak	2023	Lee Wee Ting EC 012	Leaf (dried)	cult. (farm)	24T23	5,710,314	2,878,667	50.7	330	256,128	89.0
Coffea liberica × *C. dewevrei*	Costa Rica	2019	Hernandez/ Coffea Diversa 1872	Seed (comm.)	cult.	24T25	3,031,860	1,592,959	52.7	333	256,443	89.2
Coffea liberica × *C. dewevrei*	India	2024	PBG 157	Leaf (dried)	cult.	24L84	10,960,104	1,806,510	16.5	319	245,706	85.4
**Taxon (< 10% *C. dewevrei* admixture)**	**Country**	**Year of collection**	**Collector name and number**	**Material type**	**Cult. (C) or wild (W)**	**DNA Library code**	**No. of quality-filtered paired reads**	**No. of reads on target**	**Enrichment efficiency (% reads on target)**	**No. of genes assembled at 50% [[for 353 genes]**	**Total bp recovered [for 353 genes]**	**Efficiency recovery (% bp recovered compared to reference)**
Coffea liberica × *C. dewevrei*	Indonesia	2023	Lee Wee Ting EC 001	Seed (comm.)	cult.	24L47	14,330,964	7,857,766	54.2	327	258,834	90.0
Coffea liberica × *C. dewevrei*	Peninsular Malaysia	2024	Lee Wee Ting EC 014	Leaf (dried)	cult. (farm)	24T83	4,927,800	1,167,875	23.9	330	254,817	88.6
Coffea liberica × *C. dewevrei*	Peninsular Malaysia	2024	Lee Wee Ting EC 016	Leaf (dried)	cult. (farm)	24T85	8,474,052	1,790,938	22.5	332	253,182	88.0
Coffea liberica × *C. dewevrei*	Sarawak	2023	Lee Wee Ting LBC 001	Leaf (dried)	cult. (farm)	24L20	6,933,772	2,082,814	29.9	320	244,224	84.9
Coffea liberica × *C. dewevrei*	Sarawak	2023	Lee Wee Ting LBC 004	Leaf (dried)	cult. (farm)	24L23	3,233,448	1,161,306	35.6	317	242,577	84.3
Coffea liberica × *C. dewevrei*	Sarawak	2023	Lee Wee Ting LBC 010	Leaf (dried)	cult. (farm)	24L28	6,934,922	4,263,599	60.4	331	254,364	88.4
Coffea liberica × *C. dewevrei*	Sarawak	2023	Lee Wee Ting LBC 012	Leaf (dried)	cult. (farm)	24L30	6,605,706	3,896,675	58.0	330	259,638	90.3
Coffea liberica × *C. dewevrei*	Sarawak	2023	Lee Wee Ting LBC 019	Leaf (dried)	cult. (farm)	24L37	6,969,008	3,343,456	47.4	333	258,060	89.7
Coffea liberica × *C. dewevrei*	Sarawak	2023	Lee Wee Ting LBC 027	Leaf (dried)	cult. (farm)	24L45	11,701,362	6,122,701	51.6	334	258,231	89.8
Coffea liberica × *C. dewevrei*	Sarawak	2023	SLG 3	Leaf (dried)	cult. (farm)	24L68	7,921,106	2,686,200	33.7	326	252,141	87.7
Coffea liberica × *C. dewevrei*	Sarawak	2023	SLG 5	Leaf (dried)	cult. (farm)	24L70	6,143,634	1,859,981	30.1	324	251,487	87.4

Coffea liberica × *C. dewevrei*	Sarawak	2024	Jitam ARC SL 14	Leaf (dried)	cult. (farm)	24W62	6,703,202	3,202,187	47.8	338	256,731	89.3

Supplementary Table S2. Admixture percentages for *K = 2*

See Table S1 for accession and sequencing information.

Sample_ID	Pop_ID	K1 %	K2 %	Taxon	Country	Cultivated or wild
23C02	1	100%	0.0%	*C. dewevrei*	Sierra Leone	Cult.
23C04	1	100%	0.0%	*C. dewevrei*	Uganda	Cult.
23C05	1	100%	0.0%	*C. dewevrei*	Uganda	Cult.
23C14	1	99.9%	0.1%	*C. dewevrei*	CAR	Wild
23C18	1	100%	0.0%	*C. dewevrei*	CAR	Wild
23D44	1	100%	0.0%	*C. dewevrei*	DRC	Wild
23D49	1	97.8%	**2.2%**	*C. dewevrei*	Costa Rica	Cult.
23D50	1	99.9%	0.1%	*C. dewevrei*	Sierra Leone	Cult.
23D51	1	100%	0.0%	*C. dewevrei*	Guinea	Cult.
23D58	1	98.9%	**1.1%**	*C. dewevrei*	Hawaii	Cult.
23D59	1	96.6%	**3.4%**	*C. dewevrei*	Hawaii	Cult.
23D63	1	99.8%	0.2%	*C. dewevrei*	South Sudan	Wild
23D65	1	99.9%	0.1%	*C. dewevrei*	DRC	Wild
23D66	1	100%	0.0%	*C. dewevrei*	CAR	Wild
23D68	1	99.9%	0.1%	*C. dewevrei*	CAR	Wild
23F63	1	100%	0.0%	*C. dewevrei*	Sierra Leone	Cult.
23F66	1	100%	0.0%	*C. dewevrei*	Sierra Leone	Cult.
23F67	1	100%	0.0%	*C. dewevrei*	Sierra Leone	Cult.
23F68	1	99.0%	**1.0%**	*C. dewevrei*	Sierra Leone	Cult.
23H26	1	100%	0.0%	*C. dewevrei*	Uganda	Wild
23H28	1	100%	0.0%	*C. dewevrei*	South Sudan	Wild
23H78	1	99.9%	0.1%	*C. dewevrei*	DRC	Wild
23H83	1	100%	0.0%	*C. dewevrei*	Costa Rica	Cult.
23D56	1	100%	0.0%	*C. dewevrei*	Costa Rica	Cult.
23H75	1	100%	0.0%	*C. dewevrei*	Costa Rica	Cult.

23D53	1	100%	0.0%	*C. dewevrei*	Costa Rica	Cult.
23D57	1	100%	0.0%	*C. dewevrei*	Costa Rica	Cult.
23C01	1	100%	0.0%	*C. dewevrei*	Costa Rica	Cult.
23D54	1	100%	0.0%	*C. dewevrei*	Costa Rica	Cult.
24L56	1	98.1%	**1.9%**	*C. dewevrei*	India	Cult.
24T19	1	99.9%	0.1%	*C. dewevrei*	Vietnam	Cult.
24L82	1	98.7%	**1.3%**	*C. dewevrei*	India	Cult.
23D46	2	0.0%	100.0%	*C. liberica*	Ivory Coast	Wild
23D52	2	**0.7%**	99.3%	*C. liberica*	Peninsular Malayasia	Cult.
23H29	2	0.1%	99.9%	*C. liberica*	Sierra Leone	Wild
23H71	2	0.0%	100.0%	*C. liberica*	Madagascar	Cult.
23H72	2	0.0%	100.0%	*C. liberica*	Madagascar	Cult.
23H84	2	0.1%	99.9%	*C. liberica*	Sri Lanka	Cult.
23H85	2	0.0%	100.0%	*C. liberica*	Nigeria	Cult./Wild.
24L32	2	0.0%	100.0%	*C. liberica*	Sarawak	Cult.
24L41	2	0.0%	100.0%	*C. liberica*	Sarawak	Cult.
24L43	2	0.0%	100.0%	*C. liberica*	Sarawak	Cult.
24L80	2	0.2%	99.8%	*C. liberica*	Peninsular Malaysia	Cult.
24L81	2	0.0%	100.0%	*C. liberica*	Sarawak	Cult.
24T20	2	0.0%	100.0%	*C. liberica*	Sarawak	Cult.
24T83	2	0.0%	100.0%	*C. liberica*	Peninsular Malaysia	Cult.
24T84	2	0.0%	100.0%	*C. liberica*	Peninsular Malaysia	Cult.
24T86	2	0.0%	100.0%	*C. liberica*	Peninsular Malaysia	Cult.
25C16	2	0.0%	100.0%	*C. liberica*	India	Cult.
25C35	2	0.0%	100.0%	*C. liberica*	Peninsular Malaysia	Cult.
25C37	2	0.0%	100.0%	*C. liberica*	Sierra Leone	Cult.
25C38	2	**1.9%**	98.1%	*C. liberica*	Dominica	Cult.
25C83	2	**1.2%**	98.8%	*C. liberica*	Cameroon	Cult.
23H74	3	64.1%	35.9%	*C. liberica* × *C. dewevrei*	Costa Rica	Cult.
23H86	3	53.4%	46.6%	*C. liberica* × *C. dewevrei*	Uganda	Cult.

23H87	3	71.8%	28.2%	*C. liberica* × *C. dewevrei*	Costa Rica	Cult.
24L20	3	7.4%	92.6%	*C. liberica* × *C. dewevrei*	Sarawak	Cult.
24L21	3	14.8%	85.2%	*C. liberica* × *C. dewevrei*	Sarawak	Cult.
24L22	3	32.8%	67.2%	*C. liberica* × *C. dewevrei*	Sarawak	Cult.
24L23	3	5.8%	94.2%	*C. liberica* × *C. dewevrei*	Sarawak	Cult.
24L24	3	41.9%	58.1%	*C. liberica* × *C. dewevrei*	Sarawak	Cult.
24L25	3	40.3%	59.7%	*C. liberica* × *C. dewevrei*	Sarawak	Cult.
24L26	3	26.2%	73.8%	*C. liberica* × *C. dewevrei*	Sarawak	Cult.
24L27	3	13.3%	86.7%	*C. liberica* × *C. dewevrei*	Sarawak	Cult.
24L28	3	4.2%	95.8%	*C. liberica* × *C. dewevrei*	Sarawak	Cult.
24L29	3	31.4%	68.6%	*C. liberica* × *C. dewevrei*	Sarawak	Cult.
24L30	3	8.8%	91.2%	*C. liberica* × *C. dewevrei*	Sarawak	Cult.
24L31	3	43.1%	56.9%	*C. liberica* × *C. dewevrei*	Sarawak	Cult.
24L33	3	28.7%	71.3%	*C. liberica* × *C. dewevrei*	Sarawak	Cult.
24L34	3	27.3%	72.7%	*C. liberica* × *C. dewevrei*	Sarawak	Cult.
24L35	3	17.7%	82.3%	*C. liberica* × *C. dewevrei*	Sarawak	Cult.
24L36	3	52.1%	47.9%	*C. liberica* × *C. dewevrei*	Sarawak	Cult.
24L37	3	5.9%	94.1%	*C. liberica* × *C. dewevrei*	Sarawak	Cult.
24L38	3	10.4%	89.6%	*C. liberica* × *C. dewevrei*	Sarawak	Cult.
24L39	3	15.9%	84.1%	*C. liberica* × *C. dewevrei*	Sarawak	Cult.
24L40	3	15.8%	84.2%	*C. liberica* × *C. dewevrei*	Sarawak	Cult.
24L42	3	20.2%	79.8%	*C. liberica* × *C. dewevrei*	Sarawak	Cult.
24L44	3	22.6%	77.4%	*C. liberica* × *C. dewevrei*	Sarawak	Cult.
24L45	3	6.9%	93.1%	*C. liberica* × *C. dewevrei*	Sarawak	Cult.
24L46	3	12.1%	87.9%	*C. liberica* × *C. dewevrei*	Sarawak	Cult.
24L47	3	90.4%	9.6%	*C. liberica* × *C. dewevrei*	Indonesia	Cult.
24L48	3	51.9%	48.1%	*C. liberica* × *C. dewevrei*	Indonesia	Cult.
24L53	3	83.7%	16.3%	*C. liberica* × *C. dewevrei*	India	Cult.
24L59	3	81.3%	18.7%	*C. liberica* × *C. dewevrei*	India	Cult.
24L66	3	25.0%	75.0%	*C. liberica* × *C. dewevrei*	Sarawak	Cult.

24L67	3	15.2%	84.8%	*C. liberica* × *C. dewevrei*	Sarawak	Cult.
24L68	3	7.2%	92.8%	*C. liberica* × *C. dewevrei*	Sarawak	Cult.
24L69	3	17.4%	82.6%	*C. liberica* × *C. dewevrei*	Sarawak	Cult.
24L70	3	4.7%	95.3%	*C. liberica* × *C. dewevrei*	Sarawak	Cult.
24L71	3	16.4%	83.6%	*C. liberica* × *C. dewevrei*	Sarawak	Cult.
24L72	3	59.9%	40.1%	*C. liberica* × *C. dewevrei*	Sarawak	Cult.
24L73	3	51.1%	48.9%	*C. liberica* × *C. dewevrei*	Sarawak	Cult.
24L74	3	11.4%	88.6%	*C. liberica* × *C. dewevrei*	Sarawak	Cult.
24L75	3	51.4%	48.6%	*C. liberica* × *C. dewevrei*	Sarawak	Cult.
24L76	3	22.1%	77.9%	*C. liberica* × *C. dewevrei*	Sarawak	Cult.
24L77	3	14.1%	85.9%	*C. liberica* × *C. dewevrei*	Sarawak	Cult.
24L78	3	13.6%	86.4%	*C. liberica* × *C. dewevrei*	Indonesia	Cult.
24L79	3	85.7%	14.3%	*C. liberica* × *C. dewevrei*	Indonesia	Cult.
24L83	3	89.8%	10.2%	*C. liberica* × *C. dewevrei*	India	Cult.
24L84	3	34.8%	65.2%	*C. liberica* × *C. dewevrei*	India	Cult.
24T18	3	78.0%	22.0%	*C. liberica* × *C. dewevrei*	Costa Rica	Cult.
24T21	3	28.2%	71.8%	*C. liberica* × *C. dewevrei*	Sarawak	Cult.
24T22	3	10.2%	89.8%	*C. liberica* × *C. dewevrei*	Sarawak	Cult.
24T23	3	39.7%	60.3%	*C. liberica* × *C. dewevrei*	Sarawak	Cult.
24T25	3	69.2%	30.8%	*C. liberica* × *C. dewevrei*	Costa Rica	Cult.
24T82	3	8.6%	91.4%	*C. liberica* × *C. dewevrei*	Peninsular Malaysia	Cult.
24T85	3	4.4%	95.6%	*C. liberica* × *C. dewevrei*	Peninsular Malaysia	Cult.
24W62	3	2.8%	97.2%	*C. liberica* × *C. dewevrei*	Sarawak	Cult.
25C24	3	85.8%	14.2%	*C. liberica* × *C. dewevrei*	India	Cult.
25C25	3	81.5%	18.5%	*C. liberica* × *C. dewevrei*	India	Cult.
25C26	3	80.9%	19.1%	*C. liberica* × *C. dewevrei*	India	Cult.
25C27	3	74.0%	26.0%	*C. liberica* × *C. dewevrei*	India	Cult.
25C28	3	67.2%	32.8%	*C. liberica* × *C. dewevrei*	India	Cult.

Supplementary Table S3. Admixture percentages for *K* = 3 *and K* = 4.
See Table S1 for accession and sequencing information.

K = 3

Sample_ID	Pop_ID	K1 %	K2 %	K3 %	Taxon	Country	Cultivated or wild
23C02	1	99.5%	0.4%	0.0%	*C. dewevrei*	Sierra Leone	Cult.
23C04	1	99.9%	0.1%	0.0%	*C. dewevrei*	Uganda	Cult.
23C05	1	99.4%	0.6%	0.0%	*C. dewevrei*	Uganda	Cult.
23C14	1	99.8%	0.1%	0.1%	*C. dewevrei*	CAR	Wild
23C18	1	99.0%	1.0%	0.0%	*C. dewevrei*	CAR	Wild
23D44	1	98.8%	1.2%	0.0%	*C. dewevrei*	DRC	Wild
23D49	1	96.8%	1.0%	2.2%	*C. dewevrei*	Costa Rica	Cult.
23D50	1	99.8%	0.1%	0.2%	*C. dewevrei*	Sierra Leone	Cult.
23D51	1	98.8%	1.2%	0.0%	*C. dewevrei*	Guinea	Cult.
23D58	1	97.7%	1.3%	1.1%	*C. dewevrei*	Hawaii	Cult.
23D59	1	95.8%	0.9%	3.3%	*C. dewevrei*	Hawaii	Cult.
23D63	1	97.3%	2.5%	0.2%	*C. dewevrei*	South Sudan	Wild
23D65	1	98.9%	1.0%	0.0%	*C. dewevrei*	DRC	Wild
23D66	1	98.8%	1.2%	0.0%	*C. dewevrei*	CAR	Wild
23D68	1	98.3%	1.6%	0.1%	*C. dewevrei*	CAR	Wild
23F63	1	99.4%	0.6%	0.0%	*C. dewevrei*	Sierra Leone	Cult.
23F66	1	100.0%	0.0%	0.0%	*C. dewevrei*	Sierra Leone	Cult.
23F67	1	100.0%	0.0%	0.0%	*C. dewevrei*	Sierra Leone	Cult.
23F68	1	98.8%	0.1%	1.1%	*C. dewevrei*	Sierra Leone	Cult.
23H26	1	97.2%	2.8%	0.0%	*C. dewevrei*	Uganda	Wild
23H28	1	98.4%	1.6%	0.0%	*C. dewevrei*	South Sudan	Wild

23H78	1	98.1%	1.8%	0.1%	*C. dewevrei*	DRC	Wild
23D46	2	0.0%	1.3%	98.7%	*C. liberica*	Ivory Coast	Wild
23D52	2	0.8%	0.1%	99.1%	*C. liberica*	Peninsular Malayasia	Cult.
23H29	2	0.0%	1.2%	98.7%	*C. liberica*	Sierra Leone	Wild
23H71	2	0.0%	0.0%	100.0%	*C. liberica*	Madagascar	Cult.
23H72	2	0.0%	0.1%	99.9%	*C. liberica*	Madagascar	Cult.
23H84	2	0.2%	0.1%	99.8%	*C. liberica*	Sri Lanka	Cult.
23H85	2	0.0%	0.0%	100.0%	*C. liberica*	Nigeria	Wild.
24L32	2	0.0%	0.1%	99.9%	*C. liberica*	Sarawak	Cult.
24L41	2	0.0%	0.0%	100.0%	*C. liberica*	Sarawak	Cult.
24L43	2	0.0%	0.0%	100.0%	*C. liberica*	Sarawak	Cult.
24L80	2	0.2%	0.2%	99.6%	*C. liberica*	Peninsular Malaysia	Cult.
24L81	2	0.0%	0.0%	99.9%	*C. liberica*	Sarawak	Cult.
24T20	2	0.0%	0.1%	99.9%	*C. liberica*	Sarawak	Cult.
24T83	2	0.0%	0.0%	99.9%	*C. liberica*	Peninsular Malaysia	Cult.
24T84	2	0.0%	0.0%	100.0%	*C. liberica*	Peninsular Malaysia	Cult.
24T86	2	0.0%	0.0%	99.9%	*C. liberica*	Peninsular Malaysia	Cult.
25C16	2	0.0%	0.0%	99.9%	*C. liberica*	India	Cult.
25C35	2	0.0%	0.0%	100.0%	*C. liberica*	Peninsular Malaysia	Cult.
25C37	2	0.0%	0.0%	100.0%	*C. liberica*	Sierra Leone	Cult.
25C38	2	2.1%	0.1%	97.8%	*C. liberica*	Dominica	Cult.
25C83	2	1.1%	0.5%	98.4%	*C. liberica*	Cameroon	Cult.
23H74	3	60.5%	6.5%	32.9%	*C. liberica* × *C. dewevrei*	Costa Rica	Cult.
23H83	3	99.2%	0.8%	0.0%	*C. liberica* × *C. dewevrei*	Costa Rica	Cult.
23H86	3	51.7%	2.0%	46.4%	*C. liberica* × *C. dewevrei*	Uganda	Cult.
23H87	3	70.6%	0.9%	28.5%	*C. liberica* × *C. dewevrei*	Costa Rica	Cult.
24L20	3	5.8%	3.3%	90.9%	*C. liberica* × *C. dewevrei*	Sarawak	Cult.
24L21	3	14.4%	1.0%	84.6%	*C. liberica* × *C. dewevrei*	Sarawak	Cult.
24L22	3	27.9%	6.2%	65.9%	*C. liberica* × *C. dewevrei*	Sarawak	Cult.
24L23	3	3.1%	4.8%	92.2%	*C. liberica* × *C. dewevrei*	Sarawak	Cult.

24L24	3	38.7%	4.0%	57.3%	*C. liberica* × *C. dewevrei*	Sarawak	Cult.
24L25	3	31.6%	13.0%	55.4%	*C. liberica* × *C. dewevrei*	Sarawak	Cult.
24L26	3	23.6%	3.9%	72.5%	*C. liberica* × *C. dewevrei*	Sarawak	Cult.
24L27	3	10.6%	4.9%	84.6%	*C. liberica* × *C. dewevrei*	Sarawak	Cult.
24L28	3	3.6%	1.7%	94.7%	*C. liberica* × *C. dewevrei*	Sarawak	Cult.
24L29	3	17.5%	20.9%	61.6%	*C. liberica* × *C. dewevrei*	Sarawak	Cult.
24L30	3	6.2%	4.1%	89.8%	*C. liberica* × *C. dewevrei*	Sarawak	Cult.
24L31	3	37.6%	8.0%	54.4%	*C. liberica* × *C. dewevrei*	Sarawak	Cult.
24L33	3	21.9%	10.9%	67.2%	*C. liberica* × *C. dewevrei*	Sarawak	Cult.
24L34	3	18.7%	14.1%	67.3%	*C. liberica* × *C. dewevrei*	Sarawak	Cult.
24L35	3	14.2%	4.7%	81.1%	*C. liberica* × *C. dewevrei*	Sarawak	Cult.
24L36	3	45.6%	8.7%	45.7%	*C. liberica* × *C. dewevrei*	Sarawak	Cult.
24L37	3	4.3%	3.8%	91.9%	*C. liberica* × *C. dewevrei*	Sarawak	Cult.
24L38	3	8.0%	3.4%	88.6%	*C. liberica* × *C. dewevrei*	Sarawak	Cult.
24L39	3	5.1%	18.1%	76.8%	*C. liberica* × *C. dewevrei*	Sarawak	Cult.
24L40	3	5.7%	17.1%	77.3%	*C. liberica* × *C. dewevrei*	Sarawak	Cult.
24L42	3	11.4%	13.3%	75.3%	*C. liberica* × *C. dewevrei*	Sarawak	Cult.
24L44	3	9.4%	17.2%	73.4%	*C. liberica* × *C. dewevrei*	Sarawak	Cult.
24L45	3	4.7%	4.7%	90.6%	*C. liberica* × *C. dewevrei*	Sarawak	Cult.
24L46	3	9.8%	4.1%	86.1%	*C. liberica* × *C. dewevrei*	Sarawak	Cult.
24L47	3	87.1%	6.1%	6.8%	*C. liberica* × *C. dewevrei*	Indonesia	Cult.
24L48	3	41.2%	20.2%	38.6%	*C. liberica* × *C. dewevrei*	Indonesia	Cult.
24L53	3	82.3%	2.0%	15.6%	*C. liberica* × *C. dewevrei*	India	Cult.
24L56	3	96.4%	1.9%	1.7%	*C. dewevrei*	India	Cult.
24L59	3	80.5%	0.5%	19.0%	*C. liberica* × *C. dewevrei*	India	Cult.
24L66	3	10.0%	18.9%	71.1%	*C. liberica* × *C. dewevrei*	Sarawak	Cult.
24L67	3	1.7%	22.3%	76.0%	*C. liberica* × *C. dewevrei*	Sarawak	Cult.
24L68	3	5.7%	3.6%	90.8%	*C. liberica* × *C. dewevrei*	Sarawak	Cult.
24L69	3	7.0%	16.3%	76.7%	*C. liberica* × *C. dewevrei*	Sarawak	Cult.
24L70	3	3.2%	2.7%	94.1%	*C. liberica* × *C. dewevrei*	Sarawak	Cult.

24L71	3	14.1%	3.4%	82.5%	*C. liberica* × *C. dewevrei*	Sarawak	Cult.
24L72	3	51.8%	12.2%	36.0%	*C. liberica* × *C. dewevrei*	Sarawak	Cult.
24L73	3	41.9%	12.5%	45.6%	*C. liberica* × *C. dewevrei*	Sarawak	Cult.
24L74	3	11.6%	0.1%	88.3%	*C. liberica* × *C. dewevrei*	Sarawak	Cult.
24L75	3	49.3%	2.5%	48.2%	*C. liberica* × *C. dewevrei*	Sarawak	Cult.
24L76	3	15.3%	10.1%	74.6%	*C. liberica* × *C. dewevrei*	Sarawak	Cult.
24L77	3	1.6%	21.2%	77.2%	*C. liberica* × *C. dewevrei*	Sarawak	Cult.
24L78	3	2.8%	18.6%	78.5%	*C. liberica* × *C. dewevrei*	Indonesia	Cult.
24L79	3	83.2%	4.2%	12.6%	*C. liberica* × *C. dewevrei*	Indonesia	Cult.
24L82	3	97.3%	1.5%	1.2%	*C. dewevrei*	India	Cult.
24L83	3	88.0%	1.8%	10.2%	*C. liberica* × *C. dewevrei*	India	Cult.
24L84	3	33.5%	1.8%	64.7%	*C. liberica* × *C. dewevrei*	India	Cult.
24T18	3	77.0%	0.8%	22.2%	*C. liberica* × *C. dewevrei*	Costa Rica	Cult.
24T19	3	99.5%	0.4%	0.1%	*C. dewevrei*	Vietnam	Cult.
24T21	3	24.3%	5.4%	70.2%	*C. liberica* × *C. dewevrei*	Sarawak	Cult.
24T22	3	7.9%	3.3%	88.8%	*C. liberica* × *C. dewevrei*	Sarawak	Cult.
24T23	3	31.5%	11.9%	56.6%	*C. liberica* × *C. dewevrei*	Sarawak	Cult.
24T25	3	66.5%	4.5%	29.0%	*C. liberica* × *C. dewevrei*	Costa Rica	Cult.
24T82	3	9.0%	0.0%	91.0%	*C. liberica* × *C. dewevrei*	Peninsular Malaysia	Cult.
24T85	3	4.6%	0.5%	94.9%	*C. liberica* × *C. dewevrei*	Peninsular Malaysia	Cult.
24W62	3	3.0%	0.0%	96.9%	*C. liberica* × *C. dewevrei*	Sarawak	Cult.
25C24	3	85.3%	0.2%	14.5%	*C. liberica* × *C. dewevrei*	India	Cult.
25C25	3	81.0%	0.2%	18.9%	*C. liberica* × *C. dewevrei*	India	Cult.
25C26	3	80.4%	0.1%	19.5%	*C. liberica* × *C. dewevrei*	India	Cult.
25C27	3	72.6%	1.5%	25.9%	*C. liberica* × *C. dewevrei*	India	Cult.
25C28	3	66.7%	0.1%	33.2%	*C. liberica* × *C. dewevrei*	India	Cult.

K = 4

Sample_ID	Pop_ID	K1 %	K2 %	K3 %	K4 %	Taxon	Country	Cultivated or wild
23C02	1	0.0%	0.0%	23.9%	76.1%	*C. dewevrei*	Sierra Leone	Cult.
23C04	1	0.0%	0.0%	77.5%	22.5%	*C. dewevrei*	Uganda	Cult.
23C05	1	0.0%	0.0%	72.7%	27.3%	*C. dewevrei*	Uganda	Cult.
23C14	1	0.1%	0.0%	68.0%	31.9%	*C. dewevrei*	CAR	Wild
23C18	1	0.0%	0.0%	34.9%	65.1%	*C. dewevrei*	CAR	Wild
23D44	1	0.0%	0.0%	30.9%	69.0%	*C. dewevrei*	DRC	Wild
23D49	1	0.0%	2.0%	55.3%	42.8%	*C. dewevrei*	Costa Rica	Cult.
23D50	1	0.0%	0.1%	21.3%	78.6%	*C. dewevrei*	Sierra Leone	Cult.
23D51	1	0.0%	0.0%	42.7%	57.3%	*C. dewevrei*	Guinea	Cult.
23D58	1	0.0%	0.8%	64.3%	34.9%	*C. dewevrei*	Hawaii	Cult.
23D59	1	0.0%	3.4%	79.3%	17.3%	*C. dewevrei*	Hawaii	Cult.
23D63	1	0.0%	0.1%	26.8%	73.1%	*C. dewevrei*	South Sudan	Wild
23D65	1	0.0%	0.0%	58.9%	41.1%	*C. dewevrei*	DRC	Wild
23D66	1	0.0%	0.0%	33.4%	66.6%	*C. dewevrei*	CAR	Wild
23D68	1	0.0%	0.0%	37.4%	62.5%	*C. dewevrei*	CAR	Wild
23F63	1	0.0%	0.0%	27.9%	72.0%	*C. dewevrei*	Sierra Leone	Cult.
23F66	1	0.0%	0.0%	16.5%	83.5%	*C. dewevrei*	Sierra Leone	Cult.
23F67	1	0.0%	0.0%	16.6%	83.3%	*C. dewevrei*	Sierra Leone	Cult.
23F68	1	0.0%	0.3%	15.0%	84.6%	*C. dewevrei*	Sierra Leone	Cult.
23H26	1	0.0%	0.0%	16.8%	83.2%	*C. dewevrei*	Uganda	Wild
23H28	1	0.0%	0.0%	23.9%	76.0%	*C. dewevrei*	South Sudan	Wild
23H78	1	0.0%	0.1%	24.2%	75.7%	*C. dewevrei*	DRC	Wild
23D46	2	1.0%	98.9%	0.0%	0.1%	*C. liberica*	Ivory Coast	Wild
23D52	2	0.0%	99.2%	0.0%	0.7%	*C. liberica*	Peninsular Malayasia	Cult.
23H29	2	1.1%	98.9%	0.0%	0.0%	*C. liberica*	Sierra Leone	Wild

23H71	2	0.0%	100.0%	0.0%	0.0%	*C. liberica*	Madagascar	Cult.
23H72	2	0.0%	100.0%	0.0%	0.0%	*C. liberica*	Madagascar	Cult.
23H84	2	0.0%	99.8%	0.1%	0.1%	*C. liberica*	Sri Lanka	Cult.
23H85	2	0.0%	99.9%	0.0%	0.0%	*C. liberica*	Nigeria	Wild.
24L32	2	0.1%	99.9%	0.0%	0.0%	*C. liberica*	Sarawak	Cult.
24L41	2	0.0%	100.0%	0.0%	0.0%	*C. liberica*	Sarawak	Cult.
24L43	2	0.0%	100.0%	0.0%	0.0%	*C. liberica*	Sarawak	Cult.
24L80	2	0.3%	99.6%	0.1%	0.1%	*C. liberica*	Peninsular Malaysia	Cult.
24L81	2	0.0%	99.9%	0.0%	0.0%	*C. liberica*	Sarawak	Cult.
24T20	2	0.1%	99.9%	0.0%	0.0%	*C. liberica*	Sarawak	Cult.
24T83	2	0.0%	99.9%	0.0%	0.0%	*C. liberica*	Peninsular Malaysia	Cult.
24T84	2	0.0%	100.0%	0.0%	0.0%	*C. liberica*	Peninsular Malaysia	Cult.
24T86	2	0.0%	99.9%	0.0%	0.0%	*C. liberica*	Peninsular Malaysia	Cult.
25C16	2	0.0%	99.9%	0.0%	0.0%	*C. liberica*	India	Cult.
25C35	2	0.0%	100.0%	0.0%	0.0%	*C. liberica*	Peninsular Malaysia	Cult.
25C37	2	0.0%	100.0%	0.0%	0.0%	*C. liberica*	Sierra Leone	Cult.
25C38	2	0.2%	97.9%	0.3%	1.7%	*C. liberica*	Dominica	Cult.
25C83	2	0.7%	98.3%	0.1%	0.9%	*C. liberica*	Cameroon	Cult.
23H74	3	6.3%	32.1%	44.0%	17.6%	*C. liberica* × *C. dewevrei*	Costa Rica	Cult.
23H83	3	0.0%	0.0%	42.8%	57.1%	*C. liberica* × *C. dewevrei*	Costa Rica	Cult.
23H86	3	0.2%	46.2%	47.5%	6.1%	*C. liberica* × *C. dewevrei*	Uganda	Cult.
23H87	3	0.0%	28.8%	35.6%	35.6%	*C. liberica* × *C. dewevrei*	Costa Rica	Cult.
24L20	3	3.1%	89.9%	6.9%	0.0%	*C. liberica* × *C. dewevrei*	Sarawak	Cult.
24L21	3	0.1%	83.9%	15.9%	0.1%	*C. liberica* × *C. dewevrei*	Sarawak	Cult.
24L22	3	4.4%	64.2%	31.3%	0.0%	*C. liberica* × *C. dewevrei*	Sarawak	Cult.
24L23	3	4.4%	90.7%	4.9%	0.0%	*C. liberica* × *C. dewevrei*	Sarawak	Cult.
24L24	3	1.5%	56.4%	41.9%	0.1%	*C. liberica* × *C. dewevrei*	Sarawak	Cult.
24L25	3	11.6%	52.8%	35.6%	0.0%	*C. liberica* × *C. dewevrei*	Sarawak	Cult.
24L26	3	2.5%	71.4%	26.0%	0.0%	*C. liberica* × *C. dewevrei*	Sarawak	Cult.
24L27	3	4.4%	83.2%	12.3%	0.0%	*C. liberica* × *C. dewevrei*	Sarawak	Cult.

24L28	3	1.9%	94.2%	3.9%	0.0%	*C. liberica* × *C. dewevrei*	Sarawak	Cult.
24L29	3	20.8%	56.8%	22.3%	0.0%	*C. liberica* × *C. dewevrei*	Sarawak	Cult.
24L30	3	3.3%	88.7%	8.0%	0.0%	*C. liberica* × *C. dewevrei*	Sarawak	Cult.
24L31	3	6.5%	52.7%	40.7%	0.1%	*C. liberica* × *C. dewevrei*	Sarawak	Cult.
24L33	3	10.4%	64.6%	25.0%	0.0%	*C. liberica* × *C. dewevrei*	Sarawak	Cult.
24L34	3	15.1%	63.9%	20.9%	0.0%	*C. liberica* × *C. dewevrei*	Sarawak	Cult.
24L35	3	3.1%	79.4%	17.4%	0.1%	*C. liberica* × *C. dewevrei*	Sarawak	Cult.
24L36	3	5.0%	44.5%	50.5%	0.0%	*C. liberica* × *C. dewevrei*	Sarawak	Cult.
24L37	3	5.0%	90.9%	3.7%	0.4%	*C. liberica* × *C. dewevrei*	Sarawak	Cult.
24L38	3	2.4%	87.5%	10.1%	0.0%	*C. liberica* × *C. dewevrei*	Sarawak	Cult.
24L39	3	21.6%	72.6%	5.7%	0.0%	*C. liberica* × *C. dewevrei*	Sarawak	Cult.
24L40	3	19.6%	72.7%	7.7%	0.0%	*C. liberica* × *C. dewevrei*	Sarawak	Cult.
24L42	3	15.6%	72.1%	12.3%	0.0%	*C. liberica* × *C. dewevrei*	Sarawak	Cult.
24L44	3	19.6%	69.5%	10.9%	0.1%	*C. liberica* × *C. dewevrei*	Sarawak	Cult.
24L45	3	4.7%	89.3%	6.0%	0.0%	*C. liberica* × *C. dewevrei*	Sarawak	Cult.
24L46	3	3.9%	84.8%	11.3%	0.0%	*C. liberica* × *C. dewevrei*	Sarawak	Cult.
24L47	3	5.5%	6.5%	70.2%	17.8%	*C. liberica* × *C. dewevrei*	Indonesia	Cult.
24L48	3	24.0%	35.1%	31.2%	9.6%	*C. liberica* × *C. dewevrei*	Indonesia	Cult.
24L53	3	0.6%	15.7%	51.9%	31.8%	*C. liberica* × *C. dewevrei*	India	Cult.
24L56	3	0.0%	1.1%	44.7%	54.2%	*C. liberica* × *C. dewevrei*	India	Cult.
24L59	3	0.1%	19.2%	68.0%	12.7%	*C. liberica* × *C. dewevrei*	India	Cult.
24L66	3	22.3%	66.6%	11.1%	0.0%	*C. liberica* × *C. dewevrei*	Sarawak	Cult.
24L67	3	28.9%	71.0%	0.0%	0.0%	*C. liberica* × *C. dewevrei*	Sarawak	Cult.
24L68	3	4.4%	90.0%	5.3%	0.4%	*C. liberica* × *C. dewevrei*	Sarawak	Cult.
24L69	3	20.3%	73.2%	6.3%	0.1%	*C. liberica* × *C. dewevrei*	Sarawak	Cult.
24L70	3	4.0%	93.6%	1.7%	0.7%	*C. liberica* × *C. dewevrei*	Sarawak	Cult.
24L71	3	1.8%	81.5%	16.6%	0.0%	*C. liberica* × *C. dewevrei*	Sarawak	Cult.
24L72	3	9.6%	34.4%	55.9%	0.1%	*C. liberica* × *C. dewevrei*	Sarawak	Cult.
24L73	3	8.9%	43.4%	47.7%	0.0%	*C. liberica* × *C. dewevrei*	Sarawak	Cult.
24L74	3	0.0%	87.8%	12.0%	0.2%	*C. liberica* × *C. dewevrei*	Sarawak	Cult.

24L75	3	0.1%	48.1%	51.5%	0.4%	*C. liberica* × *C. dewevrei*	Sarawak	Cult.
24L76	3	9.4%	71.8%	18.8%	0.0%	*C. liberica* × *C. dewevrei*	Sarawak	Cult.
24L77	3	27.7%	72.3%	0.0%	0.0%	*C. liberica* × *C. dewevrei*	Sarawak	Cult.
24L78	3	23.1%	74.3%	2.5%	0.1%	*C. liberica* × *C. dewevrei*	Indonesia	Cult.
24L79	3	2.9%	12.8%	70.7%	13.6%	*C. liberica* × *C. dewevrei*	Indonesia	Cult.
24L82	3	0.0%	0.6%	43.6%	55.7%	*C. liberica* × *C. dewevrei*	India	Cult.
24L83	3	0.0%	10.2%	38.8%	51.0%	*C. liberica* × *C. dewevrei*	India	Cult.
24L84	3	1.0%	64.0%	33.3%	1.7%	*C. liberica* × *C. dewevrei*	India	Cult.
24T18	3	0.0%	22.4%	33.7%	43.9%	*C. liberica* × *C. dewevrei*	Costa Rica	Cult.
24T19	3	0.0%	0.1%	92.0%	7.9%	*C. liberica* × *C. dewevrei*	Vietnam	Cult.
24T21	3	3.4%	68.7%	27.9%	0.0%	*C. liberica* × *C. dewevrei*	Sarawak	Cult.
24T22	3	2.0%	87.7%	10.2%	0.0%	*C. liberica* × *C. dewevrei*	Sarawak	Cult.
24T23	3	11.2%	53.9%	34.9%	0.1%	*C. liberica* × *C. dewevrei*	Sarawak	Cult.
24T25	3	4.2%	28.6%	47.5%	19.7%	*C. liberica* × *C. dewevrei*	Costa Rica	Cult.
24T82	3	0.0%	90.5%	9.4%	0.1%	*C. liberica* × *C. dewevrei*	Peninsular Malaysia	Cult.
24T85	3	0.1%	94.5%	5.4%	0.0%	*C. liberica* × *C. dewevrei*	Peninsular Malaysia	Cult.
24W62	3	0.0%	96.6%	3.3%	0.0%	*C. liberica* × *C. dewevrei*	Sarawak	Cult.
25C24	3	0.0%	14.6%	74.4%	11.0%	*C. liberica* × *C. dewevrei*	India	Cult.
25C25	3	0.0%	18.6%	77.9%	3.5%	*C. liberica* × *C. dewevrei*	India	Cult.
25C26	3	0.0%	19.3%	72.2%	8.5%	*C. liberica* × *C. dewevrei*	India	Cult.
25C27	3	0.2%	25.5%	73.2%	1.1%	*C. liberica* × *C. dewevrei*	India	Cult.
25C28	3	0.0%	32.9%	62.8%	4.2%	*C. liberica* × *C. dewevrei*	India	Cult.

Suppl. Table S4. Admixture percentages *K = 2* for Malaysian accessions

See Table S1 for accession and sequencing information.

Sample_ID	Pop_ID	K1 %	K2 %	Taxon	Peninsular Malaysia/ Sarawak	Farm no. (>2 accessions)
23D52	2	0.70%	99.30%	*C. liberica*	Peninsular Malaysia	
24T83	2	0.00%	100.00%	*C. liberica*	Peninsular Malaysia	
24T84	2	0.00%	100.00%	*C. liberica*	Peninsular Malaysia	
24L80	2	0.20%	99.80%	*C. liberica*	Peninsular Malaysia	
25C35	2	0.00%	100.00%	*C. liberica*	Peninsular Malaysia	
24T86	2	0.00%	100.00%	*C. liberica*	Peninsular Malaysia	
24L21	3	14.80%	85.20%	*C. liberica* × *C. dewevrei*	Sarawak	1
24L22	3	**32.80%**	67.20%	*C. liberica* × *C. dewevrei*	Sarawak	1
24L23	3	5.80%	94.20%	*C. liberica* × *C. dewevrei*	Sarawak	1
24L24	3	41.90%	58.10%	*C. liberica* × *C. dewevrei*	Sarawak	1
24L43	2	0.00%	100.00%	*C. liberica*	Sarawak	2
24L42	3	20.20%	79.80%	*C. liberica* × *C. dewevrei*	Sarawak	2
24L44	3	22.60%	77.40%	*C. liberica* × *C. dewevrei*	Sarawak	2
24L34	3	27.30%	72.70%	*C. liberica* × *C. dewevrei*	Sarawak	3
24L35	3	17.70%	82.30%	*C. liberica* × *C. dewevrei*	Sarawak	3
24L36	3	52.10%	47.90%	*C. liberica* × *C. dewevrei*	Sarawak	3
24T22	3	10.20%	89.80%	*C. liberica* × *C. dewevrei*	Sarawak	3
24T23	3	39.70%	60.30%	*C. liberica* × *C. dewevrei*	Sarawak	3
24L32	2	0.00%	100.00%	*C. liberica*	Sarawak	4
24T20	2	0.00%	100.00%	*C. liberica*	Sarawak	4
24L26	3	26.20%	73.80%	*C. liberica* × *C. dewevrei*	Sarawak	4
24L27	3	13.30%	86.70%	*C. liberica* × *C. dewevrei*	Sarawak	4
24L28	3	4.20%	95.80%	*C. liberica* × *C. dewevrei*	Sarawak	4

24L29	3	31.40%	68.60%	*C. liberica × C. dewevrei*	Sarawak	4
24L30	3	8.80%	91.20%	*C. liberica × C. dewevrei*	Sarawak	4
24L31	3	43.10%	56.90%	*C. liberica × C. dewevrei*	Sarawak	4
24L33	3	28.70%	71.30%	*C. liberica × C. dewevrei*	Sarawak	4
24L71	3	16.40%	83.60%	*C. liberica × C. dewevrei*	Sarawak	4
24L72	3	59.90%	40.10%	*C. liberica × C. dewevrei*	Sarawak	4
24L73	3	51.10%	48.90%	*C. liberica × C. dewevrei*	Sarawak	4
24T21	3	28.20%	71.80%	*C. liberica × C. dewevrei*	Sarawak	4
24L41	2	0.00%	100.00%	*C. liberica*	Sarawak	5
24L39	3	15.90%	84.10%	*C. liberica × C. dewevrei*	Sarawak	5
24L40	3	15.80%	84.20%	*C. liberica × C. dewevrei*	Sarawak	5
24L69	3	17.40%	82.60%	*C. liberica × C. dewevrei*	Sarawak	5
24L74	3	11.40%	88.60%	*C. liberica × C. dewevrei*	Sarawak	6
24L75	3	51.40%	48.60%	*C. liberica × C. dewevrei*	Sarawak	6
24L76	3	22.10%	77.90%	*C. liberica × C. dewevrei*	Sarawak	6
24L45	3	6.90%	93.10%	*C. liberica × C. dewevrei*	Sarawak	
24L46	3	12.10%	87.90%	*C. liberica × C. dewevrei*	Sarawak	
24L37	3	5.90%	94.10%	*C. liberica × C. dewevrei*	Sarawak	
24L38	3	10.40%	89.60%	*C. liberica × C. dewevrei*	Sarawak	
24L77	3	14.10%	85.90%	*C. liberica × C. dewevrei*	Sarawak	
24L67	3	15.20%	84.80%	*C. liberica × C. dewevrei*	Sarawak	
24L25	3	40.30%	59.70%	*C. liberica × C. dewevrei*	Sarawak	
24L81	2	0.00%	100.00%	*C. liberica*	Sarawak	
24L20	3	7.40%	92.60%	*C. liberica × C. dewevrei*	Sarawak	
24L66	3	25.00%	75.00%	*C. liberica × C. dewevrei*	Sarawak	
24L68	3	7.20%	92.80%	*C. liberica × C. dewevrei*	Sarawak	
24L70	3	4.70%	95.30%	*C. liberica × C. dewevrei*	Sarawak	
24T82	3	8.60%	91.40%	*C. liberica × C. dewevrei*	Peninsular Malaysia	
24T85	3	4.40%	95.60%	*C. liberica × C. dewevrei*	Peninsular Malaysia	
24W62	3	2.80%	97.20%	*C. liberica × C. dewevrei*	Sarawak	

Supplementary Table S5. Descriptive statistics and details of ANOVA and TukeyHSD test for parchment thickness, seed length, and seed width.

Descriptive statistics (Quantitative data):

Parchment (endocarp) thickness across species

C. liberica

Statistic	Var1
Nbr. of observations	29
Minimum	0.43
Maximum	0.77
1st Quartile	0.56
Median	0.59
3rd Quartile	0.63
Mean	0.59
Variance (n-1)	0.01
Standard deviation (n-1)	0.07

C. dewevrei

Statistic	Var1
Nbr. of observations	244
Minimum	0.14
Maximum	0.59
1st Quartile	0.21
Median	0.25
3rd Quartile	0.31
Mean	0.27
Variance (n-1)	0.01
Standard deviation (n-1)	0.08

C. liberica × *C. dewevrei*

Statistic	Var1
Nbr. of observations	204
Minimum	0.18
Maximum	0.77
1st Quartile	0.33
Median	0.41
3rd Quartile	0.54
Mean	0.44
Variance (n-1)	0.02
Standard deviation (n-1)	0.14

Seed length across species

C. liberica

Statistic	Var1
Nbr. of observations	467
Minimum	6.20
Maximum	18.33
1st Quartile	10.72
Median	11.80
3rd Quartile	12.80
Mean	11.85
Variance (n-1)	3.39

C. dewevrei

Statistic	Var1
Nbr. of observations	781
Minimum	4.90
Maximum	12.70
1st Quartile	7.60
Median	8.30
3rd Quartile	9.10
Mean	8.39
Variance (n-1)	1.40

C. liberica × *C. dewevrei*

Statistic	Var1
Nbr. of observations	892
Minimum	3.70
Maximum	16.30
1st Quartile	8.80
Median	10.20
3rd Quartile	11.47
Mean	10.27
Variance (n-1)	3.51

Standard deviation (n-1)	1.84		Standard deviation (n-1)	1.19		Standard deviation (n-1)	1.87

Seed width across species and hybrids

C. liberica			*C. dewevrei*			*C. liberica* × *C. dewevrei*	
Statistic	Var1		Statistic	Var1		Statistic	Var1
Nbr. of observations	467		Nbr. of observations	781		Nbr. of observations	892
Minimum	5.00		Minimum	3.40		Minimum	4.70
Maximum	11.98		Maximum	8.50		Maximum	10.03
1st Quartile	6.80		1st Quartile	5.40		1st Quartile	6.40
Median	7.40		Median	5.90		Median	7.10
3rd Quartile	8.10		3rd Quartile	6.68		3rd Quartile	7.76
Mean	7.51		Mean	6.02		Mean	7.15
Variance (n-1)	0.93		Variance (n-1)	0.78		Variance (n-1)	0.96
Standard deviation (n-1)	0.97		Standard deviation (n-1)	0.88		Standard deviation (n-1)	0.98

ANOVA and TurkeyHSD

Parchment (endocarp) thickness across species

ANOVA

	Df	Sum Sq	Mean Sq	F values	Pr(>F)
Species	2	4.922	2.4611	214.5	<2e-16***
Residuals	474	5.439	0.0115		

TukeyHSD

	diff	lwr	upr	p adj
Coffea liberica vs. *Coffea dewevrei*	0.2913124	0.2531444	0.3294805	3.34E-11
Coffea liberica × *Coffea dewevrei* vs. *Coffea dewevrei*	0.1566248	0.1318787	0.1813709	3.34E-11
Coffea liberica × *Coffea dewevrei* vs. *Coffea liberica*	-0.1346876	-0.1740479	-0.0953273	3.35E-11

Seed length across species

ANOVA

	Df	Sum Sq	Mean Sq	F values	Pr(>F)
Species	2	3637	1818.7	664.3	<2e-16***
Residuals	2137	5851	2.7		

TukeyHSD

	diff	lwr	upr	p adj
Coffea liberica-Coffea dewevrei	3.438235	3.211234	3.665236	6.4831700E-11
Coffea liberica × *Coffea dewevrei* vs. *Coffea dewevrei*	1.879111	1.68894	2.069282	6.4831700E-11
Coffea liberica × *Coffea dewevrei* vs. *Coffea liberica*	-1.559124	-1.780777	-1.337471	6.4831700E-11

Seed width across species and hybrids

ANOVA

	Df	Sum Sq	Mean Sq	F values	Pr(>F)
Species	2	817.7	408.8	460.6	<2e-16***
Residuals	2173	1896.9	0.9		

TukeyHSD

	diff	lwr	upr	p adj
Coffea liberica-Coffea dewevrei	1.4872158	1.3579615	1.61647	0.0000000
Coffea liberica × *Coffea dewevrei* vs. *Coffea dewevrei*	1.1303283	1.022045	1.2386116	0.0000000
Coffea liberica × *Coffea dewevrei* vs. *Coffea liberica*	-0.3568875	-0.4830965	-0.2306785	0.0000000

Supplementary Table S6. European Nucleotide Archive ID and file codes.
The DNA library codes are used as the identifier: accession details are provided in Table S1. Raw reads for Angiosperms353 sequence data are available at the European Nucleotide Archive (https://www.ebi.ac.uk) under project no. PRJEB78707 and PRJEB111876 [to update]

Taxon	ENA ID code	File name	DNA Library code
C. dewevrei	ERR13479603	23F66_2.fastq.gz	23F66
C. dewevrei	ERR13479603	23F66_1.fastq.gz	23F66
C. dewevrei	ERR13479598	23F68_2.fastq.gz	23F68
C. dewevrei	ERR13479598	23F68_1.fastq.gz	23F68
C. dewevrei	ERR13479590	23D67_2.fastq.gz	23D67
C. dewevrei	ERR13479590	23D67_1.fastq.gz	23D67
C. dewevrei	ERR13479589	23D44_2.fastq.gz	23D44
C. dewevrei	ERR13479589	23D44_1.fastq.gz	23D44
C. dewevrei	ERR13479587	23F63_2.fastq.gz	23F63
C. dewevrei	ERR13479587	23F63_1.fastq.gz	23F63
C. dewevrei	ERR13479586	23D51_2.fastq.gz	23D51
C. dewevrei	ERR13479586	23D51_1.fastq.gz	23D51
C. dewevrei	ERR13479578	23C18_2.fastq.gz	23C18
C. dewevrei	ERR13479578	23C18_1.fastq.gz	23C18
C. dewevrei	ERR13479566	23H28_2.fastq.gz	23H28
C. dewevrei	ERR13479566	23H28_1.fastq.gz	23H28
C. dewevrei	ERR13479553	23D49_2.fastq.gz	23D49
C. dewevrei	ERR13479553	23D49_1.fastq.gz	23D49
C. dewevrei	ERR13479537	23D66_2.fastq.gz	23D66
C. dewevrei	ERR13479537	23D66_1.fastq.gz	23D66
C. dewevrei	ERR13479524	23D68_2.fastq.gz	23D68
C. dewevrei	ERR13479524	23D68_1.fastq.gz	23D68
C. dewevrei	ERR13479507	23D59_2.fastq.gz	23D59
C. dewevrei	ERR13479507	23D59_1.fastq.gz	23D59

C. dewevrei	ERR13479502	23D58_2.fastq.gz	23D58
C. dewevrei	ERR13479502	23D58_1.fastq.gz	23D58
C. dewevrei	ERR13479495	23H78_2.fastq.gz	23H78
C. dewevrei	ERR13479495	23H78_1.fastq.gz	23H78
C. dewevrei	ERR13479491	23D50_2.fastq.gz	23D50
C. dewevrei	ERR13479491	23D50_1.fastq.gz	23D50
C. dewevrei	ERR13479483	23C04_2.fastq.gz	23C04
C. dewevrei	ERR13479483	23C04_1.fastq.gz	23C04
C. dewevrei	ERR13476975	23C05_2.fastq.gz	23C05
C. dewevrei	ERR13476975	23C05_1.fastq.gz	23C05
C. dewevrei	ERR13476967	23D63_2.fastq.gz	23D63
C. dewevrei	ERR13476967	23D63_1.fastq.gz	23D63
C. dewevrei	ERR13476956	23C14_2.fastq.gz	23C14
C. dewevrei	ERR13476956	23C14_1.fastq.gz	23C14
C. dewevrei	ERR13476954	23H26_2.fastq.gz	23H26
C. dewevrei	ERR13476954	23H26_1.fastq.gz	23H26
C. dewevrei	ERR13476843	23C02_2.fastq.gz	23C02
C. dewevrei	ERR13476843	23C02_1.fastq.gz	23C02
C. dewevrei	*ERS29737768*	*23D44_1.fastq.gz and 23D44_2.fastq.gz*	23D44
C. dewevrei	*ERS29737769*	*23D46_1.fastq.gz and 23D46_2.fastq.gz*	23D46
C. dewevrei	*ERS29737770*	*23D50_1.fastq.gz and 23D50_2.fastq.gz*	23D50
C. dewevrei	*ERS29737771*	*23D65_1.fastq.gz and 23D65_2.fastq.gz*	23D65
C. dewevrei	*ERS29737772*	*23F67_1.fastq.gz and 23F67_2.fastq.gz*	23F67
C. dewevrei	*ERS29737773*	*23H83_1.fastq.gz and 23H83_2.fastq.gz*	23H83

C. dewevrei	*ERS29737774*	*24L56_1.fastq.gz and 24L56_2.fastq.gz*	24L56
C. dewevrei	*ERS29737775*	*24L82_1.fastq.gz and 24L82_2.fastq.gz*	24L82
C. dewevrei	*ERS29737776*	*24T19_1.fastq.gz and 24T19_2.fastq.gz*	24T19
C. dewevrei	*ERS29737777*	*23D56_1.fastq.gz and 23D56_2.fastq.gz*	23D56
C. dewevrei	*ERS29737778*	*23H75_1.fastq.gz and 23H75_2.fastq.gz*	23H75
C. dewevrei	*ERS29737779*	*23D53_1.fastq.gz and 23D53_2.fastq.gz*	23D53
C. dewevrei	*ERS29737780*	*23D57_1.fastq.gz and 23D57_2.fastq.gz*	23D57
C. dewevrei	*ERS29737781*	*23C01_1.fastq.gz and 23C01_2.fastq.gz*	23C01
C. dewevrei	*ERS29737782*	*23D54_1.fastq.gz and 23D54_2.fastq.gz*	23D54
C. liberica	ERR13476897	23H71_2.fastq.gz	23H71
C. liberica	ERR13476897	23H71_1.fastq.gz	23H71
C. liberica	ERR13476890	23H72_2.fastq.gz	23H72
C. liberica	ERR13476890	23H72_1.fastq.gz	23H72
C. liberica	ERR13476875	23H84_2.fastq.gz	23H84
C. liberica	ERR13476875	23H84_1.fastq.gz	23H84
C. liberica	ERR13476852	23D52_2.fastq.gz	23D52
C. liberica	ERR13476852	23D52_1.fastq.gz	23D52
C. liberica	ERR13476847	23H85_2.fastq.gz	23H85
C. liberica	ERR13476847	23H85_1.fastq.gz	23H85
C. liberica	ERR13476841	23H29_2.fastq.gz	23H29
C. liberica	ERR13476841	23H29_1.fastq.gz	23H29

C. liberica	*ERS29737783*	*25C83_1.fastq.gz and 25C83_2.fastq.gz*	25C83
C. liberica	*ERS29737784*	*25C38_1.fastq.gz and 25C38_2.fastq.gz*	25C38
C. liberica	*ERS29737785*	*25C16_1.fastq.gz and 25C16_2.fastq.gz*	25C16
C. liberica	*ERS29737786*	*24L80_1.fastq.gz and 24L80_2.fastq.gz*	24L80
C. liberica	*ERS29737787*	*25C38_1.fastq.gz and 25C35_2.fastq.gz*	25C35
C. liberica	*ERS29737788*	*24T86_1.fastq.gz and 24T86_2.fastq.gz*	24T86
C. liberica	*ERS29737789*	*24T82_1.fastq.gz and 24T82_2.fastq.gz*	24T82
C. liberica	*ERS29737790*	*24T84_1.fastq.gz and 24T84_2.fastq.gz*	24T84
C. liberica	*ERS29737791*	*24L32_1.fastq.gz and 24L32_2.fastq.gz*	24L32
C. liberica	*ERS29737792*	*24L41_1.fastq.gz and 24L41_2.fastq.gz*	24L41
C. liberica	*ERS29737793*	*24L43_1.fastq.gz and 24L43_2.fastq.gz*	24L43
C. liberica	*ERS29737794*	*24L81_1.fastq.gz and 24L81_2.fastq.gz*	24L81
C. liberica	*ERS29737795*	*24T20_1.fastq.gz and 24T20_2.fastq.gz*	24T20
C. liberica	*ERS29737796*	*25C37_1.fastq.gz and 25C37_2.fastq.gz*	25C37
C. liberica × C. dewevrei	ERR13476855	23H87_2.fastq.gz	23H87
C. liberica × C. dewevrei	ERR13476855	23H87_1.fastq.gz	23H87
C. liberica × C. dewevrei	ERR13476853	23H74_2.fastq.gz	23H74
C. liberica × C. dewevrei	ERR13476853	23H74_1.fastq.gz	23H74

C. liberica × C. dewevrei	*ERS29737797*	*24L48_1.fastq.gz and 24L48_2.fastq.gz*	24L48
C. liberica × C. dewevrei	*ERS29737798*	*24L79_1.fastq.gz and 24L79_2.fastq.gz*	24L79
C. liberica × C. dewevrei	*ERS29737799*	*25C24_1.fastq.gz and 25C24_2.fastq.gz*	25C24
C. liberica × C. dewevrei	*ERS29737800*	*25C25_1.fastq.gz and 25C25_2.fastq.gz*	25C25
C. liberica × C. dewevrei	*ERS29737801*	*25C26_1.fastq.gz and 25C26_2.fastq.gz*	25C26
C. liberica × C. dewevrei	*ERS29737802*	*25C27_1.fastq.gz and 25C27_2.fastq.gz*	25C27
C. liberica × C. dewevrei	*ERS29737803*	*25C28_1.fastq.gz and 25C28_2.fastq.gz*	25C28
C. liberica × C. dewevrei	*ERS29737804*	*24L59_1.fastq.gz and 24L59_2.fastq.gz*	24L59
C. liberica × C. dewevrei	*ERS29737805*	*24L53_1.fastq.gz and 24L53_2.fastq.gz*	24L53
C. liberica × C. dewevrei	*ERS29737806*	*24L83_1.fastq.gz and 24L83_2.fastq.gz*	24L83
C. liberica × C. dewevrei	*ERS29737807*	*23H86_1.fastq.gz and 23H86_2.fastq.gz*	23H86
C. liberica × C. dewevrei	*ERS29737808*	*23H87_1.fastq.gz and 23H87_2.fastq.gz*	23H87
C. liberica × C. dewevrei	*ERS29737809*	*24L21_1.fastq.gz and 24L21_2.fastq.gz*	24L21
C. liberica × C. dewevrei	*ERS29737810*	*24L22_1.fastq.gz and 24L22_2.fastq.gz*	24L22
C. liberica × C. dewevrei	*ERS29737811*	*24L24_1.fastq.gz and 24L24_2.fastq.gz*	24L24
C. liberica × C. dewevrei	*ERS29737812*	*24L25_1.fastq.gz and 24L25_2.fastq.gz*	24L25

C. liberica × C. dewevrei	*ERS29737813*	*24L26_1.fastq.gz and 24L26_2.fastq.gz*	24L26
C. liberica × C. dewevrei	*ERS29737814*	*24L27_1.fastq.gz and 24L27_2.fastq.gz*	24L27
C. liberica × C. dewevrei	*ERS29737815*	*24L29_1.fastq.gz and 24L29_2.fastq.gz*	24L29
C. liberica × C. dewevrei	*ERS29737816*	*24L31_1.fastq.gz and 24L31_2.fastq.gz*	24L31
C. liberica × C. dewevrei	*ERS29737817*	*24L33_1.fastq.gz and 24L33_2.fastq.gz*	24L33
C. liberica × C. dewevrei	*ERS29737818*	*24L34_1.fastq.gz and 24L34_2.fastq.gz*	24L34
C. liberica × C. dewevrei	*ERS29737819*	*24L35_1.fastq.gz and 24L35_2.fastq.gz*	24L35
C. liberica × C. dewevrei	*ERS29737820*	*24L36_1.fastq.gz and 24L36_2.fastq.gz*	24L36
C. liberica × C. dewevrei	*ERS29737821*	*24L38_1.fastq.gz and 24L38_2.fastq.gz*	24L38
C. liberica × C. dewevrei	*ERS29737822*	*24L39_1.fastq.gz and 24L39_2.fastq.gz*	24L39
C. liberica × C. dewevrei	*ERS29737823*	*24L40_1.fastq.gz and 24L40_2.fastq.gz*	24L40
C. liberica × C. dewevrei	*ERS29737824*	*24L42_1.fastq.gz and 24L42_2.fastq.gz*	24L42
C. liberica × C. dewevrei	*ERS29737825*	*24L44_1.fastq.gz and 24L44_2.fastq.gz*	24L44
C. liberica × C. dewevrei	*ERS29737826*	*24L46_1.fastq.gz and 24L46_2.fastq.gz*	24L46
C. liberica × C. dewevrei	*ERS29737827*	*24L66_1.fastq.gz and 24L66_2.fastq.gz*	24L66
C. liberica × C. dewevrei	*ERS29737828*	*24L67_1.fastq.gz and 24L67_2.fastq.gz*	24L67

C. liberica × C. dewevrei	*ERS29737829*	*24L69_1.fastq.gz and 24L69_2.fastq.gz*	24L69
C. liberica × C. dewevrei	*ERS29737830*	*24L71_1.fastq.gz and 24L71_2.fastq.gz*	24L71
C. liberica × C. dewevrei	*ERS29737831*	*24L72_1.fastq.gz and 24L72_2.fastq.gz*	24L72
C. liberica × C. dewevrei	*ERS29737832*	*24L73_1.fastq.gz and 24L73_2.fastq.gz*	24L73
C. liberica × C. dewevrei	*ERS29737833*	*24L74_1.fastq.gz and 24L74_2.fastq.gz*	24L74
C. liberica × C. dewevrei	*ERS29737834*	*24L75_1.fastq.gz and 24L75_2.fastq.gz*	24L75
C. liberica × C. dewevrei	*ERS29737835*	*24L76_1.fastq.gz and 24L76_2.fastq.gz*	24L76
C. liberica × C. dewevrei	*ERS29737836*	*24L77_1.fastq.gz and 24L77_2.fastq.gz*	24L77
C. liberica × C. dewevrei	*ERS29737837*	*24L78_1.fastq.gz and 24L78_2.fastq.gz*	24L78
C. liberica × C. dewevrei	*ERS29737838*	*24T18_1.fastq.gz and 24T18_2.fastq.gz*	24T18
C. liberica × C. dewevrei	*ERS29737839*	*24T21_1.fastq.gz and 24T21_2.fastq.gz*	24T21
C. liberica × C. dewevrei	*ERS29737840*	*24T22_1.fastq.gz and 24T22_2.fastq.gz*	24T22
C. liberica × C. dewevrei	*ERS29737841*	*24T22_1.fastq.gz and 24T22_2.fastq.gz*	24T23
C. liberica × C. dewevrei	*ERS29737842*	*24T25_1.fastq.gz and 24T25_2.fastq.gz*	24T25
C. liberica × C. dewevrei	*ERS29737843*	*24L84_1.fastq.gz and 24L84_2.fastq.gz*	24L84
C. liberica × C. dewevrei	*ERS29737844*	*24L47_1.fastq.gz and 24L47_2.fastq.gz*	24L47

C. liberica × *C. dewevrei*	*ERS29737845*	*24T83_1.fastq.gz and 24T83_2.fastq.gz*	24T83
C. liberica × *C. dewevrei*	*ERS29737846*	*24T85_1.fastq.gz and 24T85_2.fastq.gz*	24T85
C. liberica × *C. dewevrei*	*ERS29737847*	*24L20_1.fastq.gz and 24L20_2.fastq.gz*	24L20
C. liberica × *C. dewevrei*	*ERS29737848*	*24L23_1.fastq.gz and 24L23_2.fastq.gz*	24L23
C. liberica × *C. dewevrei*	*ERS29737849*	*24L28_1.fastq.gz and 24L28_2.fastq.gz*	24L28
C. liberica × *C. dewevrei*	*ERS29737850*	*24L30_1.fastq.gz and 24L30_2.fastq.gz*	24L30
C. liberica × *C. dewevrei*	*ERS29737851*	*24L37_1.fastq.gz and 24L37_2.fastq.gz*	24L37
C. liberica × *C. dewevrei*	*ERS29737852*	*24L45_1.fastq.gz and 24L45_2.fastq.gz*	24L45
C. liberica × *C. dewevrei*	*ERS29737853*	*24L68_1.fastq.gz and 24L68_2.fastq.gz*	24L68
C. liberica × *C. dewevrei*	*ERS29737854*	*24L70_1.fastq.gz and 24L70_2.fastq.gz*	24L70
C. liberica × *C. dewevrei*	*ERS29737855*	*24W62_1.fastq.gz and 24W62.fastq.gz*	24W62

Table S7. List of parchment (endocarp) samples with accession details, DNA codes, admixture percentages (*K* = 2) and parchment thickness measurements.

The DNA library codes are used as the identifier: accession details are provided in Table S1.

Code	DNA code (if available)	Taxon	Admixture (at K = 2)	Mean Thickness (mm)	Country
ParASC004	23C04	*C. dewevrei*	100.0%	0.306	Uganda
ParASC007	23H28	*C. dewevrei*	100.0%	0.378	Uganda
ParASC032	24T19	*C. dewevrei*	**100.0%**	0.328	Vietnam
ParASC033	NA*	*C. dewevrei*		0.270	Vietnam
ParASC048	23D49	*C. dewevrei*	97.8%	0.227	Costa Rica
ParASC049	23D56	*C. dewevrei*	100.0%	0.260	Costa Rica
ParASC050	23H75	*C. dewevrei*	100.0%	0.235	Costa Rica
ParASC051	23D53	*C. dewevrei*	100.0%	0.328	Costa Rica
ParASC053	23D57	*C. dewevrei*	100.0%	0.199	Costa Rica
ParASC054	23C01	*C. dewevrei*	100.0%	0.231	Costa Rica
ParASC055	23D54	*C. dewevrei*	100.0%	0.266	Costa Rica
ParASC056	23H83	*C. dewevrei*	100.0%	0.208	Costa Rica
ParASC059	NA*	*C. dewevrei*		0.232	Hawaii
ParASC060	23D58	*C. dewevrei*	98.9%	0.263	Hawaii
ParASC061	23D59	*C. dewevrei*	96.6%	0.279	Hawaii
ParASC062	NA*	*C. dewevrei*		0.246	Hawaii
ParASC063	NA*	*C. dewevrei*		0.180	Costa Rica
ParASC064	NA*	*C. dewevrei*		0.246	Costa Rica
ParASC065	NA*	*C. dewevrei*		0.220	Costa Rica
ParASC066	23D59	*C. dewevrei*	96.6%	0.235	Costa Rica
ParASC068	23D55	*C. dewevrei*	100.0%	0.216	Costa Rica
ParASC071	NA*	*C. dewevrei*		0.216	Costa Rica
ParASC073	24T85	*C. dewevrei*	100.0%	0.351	Uganda
ParASC074	24T89	*C. dewevrei*	100.0%	0.398	Uganda

ParASC076	23H26	*C. dewevrei*	100.0%	0.245	Uganda
ParASC067	NA*	*C. dewevrei*		0.319	Costa Rica
ParASC069	NA*	*C. dewevrei*		0.290	Costa Rica
ParASC005	24L32	*C. liberica*	0.0%	0.583	Sarawak
ParASC012	24L41	*C. liberica*	0.0%	0.633	Sarawak
ParASC005(2)	24L81	*C. liberica*	0.0%	0.583	Sarawak
ParASC020	24L32	*C. liberica*	0.0%	0.730	Sarawak
ParASC029	24L43	*C. liberica*	0.0%	0.435	Sarawak
ParASC015	24L28	*C. liberica* × *C. dewevrei*	4.2%	0.542	Sarawak
ParASC010	24L23	*C. liberica* × *C. dewevrei*	5.8%	0.496	Sarawak
ParASC024	24L37	*C. liberica* × *C. dewevrei*	5.9%	0.657	Sarawak
ParASC037	24L70	*C. liberica* × *C. dewevrei*	4.7%	0.420	Sarawak
ParASC042	24L77	*C. liberica* × *C. dewevrei*	14.1%	0.610	Sarawak
PasASC027	NA*	*C. liberica* × *C. dewevrei*		0.402	Sarawak
ParASC028	24L71	*C. liberica* × *C. dewevrei*	16.0%	0.460	Sarawak
ParASC030	24L44	*C. liberica* × *C. dewevrei*	22.6%	0.507	Sarawak
ParASC031	24L45	*C. liberica* × *C. dewevrei*	6.9%	0.405	Sarawak
ParASC034	24L71	*C. liberica* × *C. dewevrei*	16.4%	0.470	Sarawak
ParASC035	24L72	*C. liberica* × *C. dewevrei*	59.9%	0.294	Sarawak
PasASC039	24L53	*C. liberica* × *C. dewevrei*	83.7%	0.400	India
ParASC052	23H74	*C. liberica* × *C. dewevrei*	64.1%	0.282	Costa Rica
ParASC057	23H87	*C. liberica* × *C. dewevrei*	71.8%	0.294	Costa Rica
ParASC070	24T18	*C. liberica* × *C. dewevrei*	78.0%	0.321	Costa Rica
ParASC072	24T25	*C. liberica* × *C. dewevrei*	69.2%	0.309	Costa Rica
ParASC008	24L21	*C. liberica* × *C. dewevrei*	14.8%	0.550	Sarawak
ParASC008	24L71	*C. liberica* × *C. dewevrei*	16.4%	0.470	Sarawak
ParASC008	24L21	*C. liberica* × *C. dewevrei*	14.8%	0.454	Sarawak
ParASC026	24L21	*C. liberica* × *C. dewevrei*	14.8%	0.550	Sarawak
ParASC009	24L22	*C. liberica* × *C. dewevrei*	32.8%	0.550	Sarawak
ParASC011	24L24	*C. liberica* × *C. dewevrei*	41.9%	0.617	Sarawak

ParASC013	24L26	*C. liberica* × *C. dewevrei*	26.2%	0.573	Sarawak
ParASC014	24L27	*C. liberica* × *C. dewevrei*	13.3%	0.565	Sarawak
ParASC016	24L29	*C. liberica* × *C. dewevrei*	31.4%	0.573	Sarawak
ParASC017	24L30	*C. liberica* × *C. dewevrei*	8.8%	0.677	Sarawak
ParASC018	24L31	*C. liberica* × *C. dewevrei*	43.1%	0.640	Sarawak
ParASC019	24L33	*C. liberica* × *C. dewevrei*	28.7%	0.487	Sarawak
ParASC021	24L34	*C. liberica* × *C. dewevrei*	27.3%	0.590	Sarawak
ParASC022	24L35	*C. liberica* × *C. dewevrei*	17.7%	0.690	Sarawak
ParASC023	24L36	*C. liberica* × *C. dewevrei*	52.1%	0.585	Sarawak
ParASC025	24L38	*C. liberica* × *C. dewevrei*	10.4%	0.747	Sarawak

Table S8. List of seed samples with accession details, DNA codes, admixture percentages (*K* = 2) and seed measurements.

The DNA library codes are used as the identifier: accession details are provided in Table S1.

Code	DNA code (if available)	Taxon	Admixture (at K = 2)	Mean seed width (mm)	Mean seed length (mm)	Country
F001	23D63	*C. dewevrei*	100%	6.70	8.93	South Sudan
F002	23C04	*C. dewevrei*	100%	6.48	9.62	Uganda
F003	23D51	*C. dewevrei*	100%	6.56	8.34	Guinea
F004	23H28	*C. dewevrei*	100%	6.51	8.70	South Sudan
F005	23C05	*C. dewevrei*	100%	7.10	9.92	Uganda
F006	NA*	*C. dewevrei*	100%	6.15	8.32	Uganda
F007	NA*	*C. dewevrei*	100%	6.17	10.31	Uganda
F008	23D58	*C. dewevrei*	98.9%	7.37	9.80	Hawaii
MeasASC001	23C04	*C. dewevrei*	100%	6.36	8.48	Uganda
MeasASC002	23D63	*C. dewevrei*	99.8%	5.82	8.03	South Sudan
MeasASC003	23H28	*C. dewevrei*	100%	5.44	8.04	South Sudan
MeasASC005	NA*	*C. dewevrei*	100%	5.35	7.99	South Sudan
MeasASC006	NA*	*C. dewevrei*	100%	6.12	8.38	South Sudan
MeasASC007	23C05	*C. dewevrei*	100%	7.24	9.88	Uganda
MeasASC008	NA*	*C. dewevrei*	100%	5.28	7.26	South Sudan
IND01	25C16	*C. liberica*	0.0%	7.79	12.19	Indonesia
MeasASC015	25C16	*C. liberica*	0.0%	6.93	11.01	India
MeasASC016	24T20	*C. liberica*	0.0%	7.36	10.51	Sarawak
MeasASC017	24L80	*C. liberica*	0.2%	7.20	12.05	Peninsular Malaysia
MeasASC018	24L81	*C. liberica*	0.0%	6.68	10.00	Sarawak
F009	NA*	*C. liberica*	0.0%	8.21	13.05	Uganda
F010	NA*	*C. liberica*	0.0%	8.32	14.97	Uganda
F011	NA*	*C. liberica*	0.0%	8.20	11.97	Sierra Leone
F012	NA*	*C. liberica*	0.0%	8.17	11.81	Sri Lanka
F013	NA*	*C. liberica*	0.0%	9.325	12.576	Java

F014	NA*	*C. liberica*	0.0%	8.60	11.98	Jamaica
F015	NA*	*C. liberica*	0.0%	9.33	12.58	Grenada
F016	NA*	*C. liberica*	0.0%	8.08	11.16	Seychelles
F017	NA*	*C. liberica*	0.0%	8.38	11.99	St. Lucia
F018	23H84	*C. liberica*	0.0%	9.08	15.27	Sri Lanka
F019	NA*	*C. liberica*	0.0%	8.51	12.79	Ghana
F020	NA*	*C. liberica*	0.0%	7.52	11.63	Uganda
F021	23H85	*C. liberica*	0.0%	8.58	11.96	Nigeria
F022	NA*	*C. liberica*	0.0%	8.12	11.94	Sierra Leone
F023	23D52	*C. liberica*	0.7%	7.48	13.48	Peninsular Malaysia
F025	NA*	*C. liberica*	0.0%	7.49	11.73	Ghana
S002	24L41	*C. liberica*	0.0%	8.60	12.77	Sarawak
MeasASC013	25C37	*C. liberica*	0.0%	6.96	12.27	Sierra Leone
MeasASC010	24L48	*C. liberica* × *C. dewevrei*	51.9%	6.40	8.95	Indonesia
MeasASC011	NA*	*C. liberica* × *C. dewevrei*	25.0%	7.95	12.02	Indonesia
MeasASC012	24L79	*C. liberica* × *C. dewevrei*	85.7%	6.22	8.63	Indonesia
F024	24T18	*C. liberica* × *C. dewevrei*	78.0%	7.96	10.52	Costa Rica
MeasASC014	24L47	*C. liberica* × *C. dewevrei*	90.4%	6.20	8.43	Indonesia
MeaASC009	24L36	*C. liberica* × *C. dewevrei*	52.1%	7.48	9.99	Sarawak
MeasASC019	24L77	*C. liberica* × *C. dewevrei*	14.1%	7.08	10.56	Sarawak
MeasASC020	24L29	*C. liberica* × *C. dewevrei*	31.4%	6.69	9.73	Sarawak
MeasASC021	24L77	*C. liberica* × *C. dewevrei*	31.4%	6.87	10.08	Sarawak
S001	NA*	*C. liberica* × *C. dewevrei*	15.0%	7.66	11.24	Sarawak
